# Hepatitis E in suspected acute hepatitis cases in Brazil: A comparison with hepatitis A

**DOI:** 10.1371/journal.pntd.0014834

**Published:** 2026-10-26

**Authors:** Ketti Gleyzer de Oliveira, Mariana Pinheiro Alves Vasconcelos, Julia Teixeira Ton, Silvia Naomi de Oliveira Uehara, Roberta Sitnik, Denize Ornelas Pereira Salvador de Oliveira, Layze Oliveira Castberg Sousa, Ricardo Andreotti Siqueira, Peter James Robinson, Thaís Senna de Paula Domingues, Caroline Thomas Panico, César Augusto Inoue, Maria Cecília Araripe Sucupira, Maira Marranghello Maluf, Fernanda de Mello Malta, Deyvid Amgarten, Erick Dorlass, Pedro Sebe, Arlene S. Pinto, Cirley M. D. O. Lobato, Adalgisa Ferreira, Elodie Hyppolito, Raymundo Paraná, Maria Isabel Schinoni, Edmundo Pessoa de Almeida Lopes, Magali C. Luiz, Raquel F. L. Garcia, Sabrina Sabino da Silva, Aline Satie Oba-Kuniyoshi, Dennis Armando Bertolini, Mário Reis Álvares-da-Silva, Raul Salinas Arrojo, Eduardo Emerim, Gabriela P. Coral, Paulo R. Acosta, Marcelle Lucena, Rosângela Rodrigues Teixeira, Mônica da Costa Guedes, Livia Melo Villar, Lia Lewis Ximenez, Clarice Gdalevici, Maria Cássia Jacintho Mendes-Correa, Michele Soares Gomes-Gouvea, Flair José Carrilho, Gerson Sobrinho Salvador de Oliveira, Paulo Roberto A. Ferreira, Pablo Andres Munoz Torres, Glória Selegatto, Luciana Vilas Boas Casadio, Simone Tenore, Olavo Henrique Munhoz Leite, Fernanda Fernandes Souza, Tânia Reuter, Francisco Souto, Michele Nascimento-Sales, Maria Lucia Gomes Ferraz, Ana Catharina de Seixas Santos Nastri, Ana Paula Maciel Gurski, Andréa Salomão, Bruna Emanuelle Alvarenga Fanis, Cristina Pimenta, Elton Carlos de Almeida, Flávia Moreno Alves de Souza, Gerson Fernando Mendes Pereira, Mario Peribañez Gonzalez, Raymundo Soares de Azevedo, João Renato Rebello Pinho

**Affiliations:** 1 Laboratório de Patologia Clínica e de Anatomia Patológica, Hospital Israelita Albert Einstein, São Paulo, Brazil; 2 LIM07, Instituto de Medicina Tropical, Departamento de Gastroenterologia, Hospital das Clínicas HCFMUSP, Faculdade de Medicina da Universidade de São Paulo, São Paulo, Brazil; 3 Centro de Pesquisa em Medicina Tropical, Secretaria de Estado da Saúde de Rondônia, Porto Velho, Rondônia, Brazil; 4 Hospital Dia Esterina Corsini de Campo Grande, Universidade Federal de Mato Grosso do Sul, Campo Grande, Mato Grosso do Sul, Brazil; 5 Fundação de Medicina Tropical Dr. Vieira Machado de Manaus, Secretaria de Estado da Saúde do Amazonas, Manaus, Amazonas, Brazil; 6 Unidade de Saúde de Referência de Doença de Rio Branco, Secretaria Municipal da Saúde, Rio Branco, Acre, Brazil; 7 Departamento de Medicina, Universidade Federal do Maranhão, São Luís, Maranhão, Brazil; 8 Hospital São José das Doenças Infecciosas de Fortaleza, Serviço de Transplante de Fígado do Hospital Universitário Walter Cantídio, Universidade Federal do Ceará, Fortaleza, Ceará, Brazil; 9 Hospital Universitário Professor Edgar Santos de Salvador, Faculdade de Medicina da Universidade Federal da Bahia, Salvador, Bahia, Brazil; 10 Hospital de Clínicas, Universidade Federal de Pernambuco, Recife, Pernambuco, Brazil; 11 Hospital Nereu Ramos, Secretaria de Estado da Saúde, Florianópolis, Santa Catarina, Brazil; 12 Hospital Municipal São José, Prefeitura de Joinville, Joinville, Santa Catarina, Brazil; 13 Fundação Universidade Regional de Blumenau (FURB), Blumenau, Santa Catarina, Brazil; 14 UniCesumar, Maringá, Paraná, Brazil; 15 Departamento de Análises Clínicas e Biomedicina, Universidade Estadual de Maringá, Maringá, Paraná, Brazil; 16 Hospital de Clínicas de Porto Alegre, Universidade Federal do Rio Grande do Sul, Porto Alegre, Rio Grande do Sul, Brazil; 17 Centro de Testagem e Aconselhamento Santa Marta de Porto Alegre, Prefeitura de Porto Alegre, Porto Alegre, Rio Grande do Sul, Brazil; 18 Santa Casa de Porto Alegre, Universidade Federal de Ciências da Saúde de Porto Alegre, Porto Alegre, Rio Grande do Sul, Brazil; 19 Consórcio Intermunicipal de Saúde de Pato Branco, Pato Branco, Paraná, Brazil; 20 Secretaria Municipal de Saúde de Florianópolis, Prefeitura de Florianópolis, Florianópolis, Santa Catarina, Brazil; 21 Equipe de Gastrohepatologia, Hospital Felício Rocho, Belo Horizonte, Minas Gerais, Brazil; 22 Secretaria Municipal de Saúde do Rio de Janeiro, Rio de Janeiro, Rio de Janeiro, Brazil; 23 Instituto Oswaldo Cruz, Fundação Oswaldo Cruz, Rio de Janeiro, Rio de Janeiro, Brazil; 24 Polo de Heplatites, Instituto de Assistência dos Servidores do Estado do Rio de Janeiro, Rio de Janeiro, Rio de Janeiro, Brazil; 25 Departamento de Moléstias Infecciosas e Parasitárias, Faculdade de Medicina da Universidade de São Paulo, São Paulo, Brazil; 26 Hospital Universitário de São Paulo, Faculdade de Medicina da Universidade de São Paulo, São Paulo, Brazil; 27 Escola Paulista de Medicina, Universidade Federal de São Paulo, São Paulo, Brazil; 28 Centro de Referência e Tratamento de IST AIDS, Secretaria de Estado da Saúde de São Paulo, São Paulo, Brazil; 29 Disciplina de Infectologia do Centro Universitário Faculdade de Medicina do ABC de São Paulo, São Bernardo do Campo, São Paulo, Brazil; 30 Hospital das Clínicas de Ribeirão Preto, Faculdade de Medicina de Ribeirão Preto da Universidade de São Paulo, Ribeirão Preto, São Paulo, Brazil; 31 Hospital Universitário Cassiano Antônio Moraes, Universidade Federal do Espírito Santo, Vitória, Espírito Santo, Brazil; 32 Hospital Universitário Júlio Muller, Ambulatório 3 de Infectologia de Cuiabá, Cuiabá, Mato Grosso, Brazil; 33 Universidade Cruzeiro do Sul de São Paulo, São Paulo, Brazil; 34 Departamento de HIV/Aids, Tuberculose, Hepatites Virais e Infecções Sexualmente Transmissíveis, Secretaria de Vigilância em Saúde e Ambiente do Ministério da Saúde (Dathi/SVSA/MS), Brasília, Distrito Federal, Brazil; 35 Instituto de Infectologia Emílio Ribas de São Paulo, Secretaria de Estado da Saúde, São Paulo, São Paulo, Brazil; 36 LIM01, Departamento de Patologia, Faculdade de Medicina da Universidade de São Paulo, São Paulo, Brazil; 37 LIM03, Hospital das Clínicas HCFMUSP, Faculdade de Medicina da Universidade de São Paulo, São Paulo, Brazil; Instituto de Virología Dr. José María Vanella, ARGENTINA

## Abstract

Hepatitis E virus (HEV) is an underrecognized cause of acute viral hepatitis worldwide, with growing public health relevance, particularly in settings where routine diagnostic testing is limited. Hepatitis A virus (HAV) is another epidemiologically related hepatitis virus, but few studies have directly compared these infections in Brazil. This study aimed to (1) determine the frequency of acute hepatitis E (AHE) and evaluate the contribution of HEV infection to the burden of hepatitis among patients with suspected acute disease attended in public healthcare institutions in Brazil; (2) assess the prevalence of acquired immunity to HEV and HAV; and (3) compare demographic characteristics, risk factors, and clinical-laboratory findings associated with HEV and HAV infections. We enrolled 1,721 adults (≥18 years) with suspected acute hepatitis from 16 Brazilian states between October 2019 and May 2023. Serum samples were tested for anti-HEV IgG/IgM and anti-HAV IgG/IgM by immunoassay and for HEV and HAV RNA by RT-PCR. Demographic, behavioral, clinical, and laboratory data were collected using a standardized questionnaire. Group comparisons were performed using univariate analyses and logistic regression, with statistical significance set at p < 0.05. Anti-HEV IgG was detected in 89 individuals (5.2%), whereas anti-HAV IgG was detected in 1,352 (78.6%). Anti-HEV IgG seroprevalence was higher in the South, Southeast, and Central West regions and among heterosexuals, and it increased with age, reaching 13.0% among individuals aged ≥65 years. Seropositivity was associated with markers of previous exposure or immunity, including HAV, HBV, *Leptospira*, and *Toxoplasma*. A total of 124 acute hepatitis cases were identified: 16 (12.9%) AHE and 108 (87.1%) acute hepatitis A (AHA). Compared with AHA cases, AHE cases occurred in older individuals and were more frequent among women and heterosexual participants. All AHA cases occurred in urban areas, whereas AHE cases were observed in both urban and rural settings. AHA cases showed higher aminotransferase levels and more frequent jaundice than AHE cases. HEV accounts for a small but distinct proportion of suspected acute hepatitis cases in Brazil and shows epidemiological and clinical characteristics that differ from HAV. Incorporating HEV testing into routine diagnostic algorithms may improve etiological clarification and surveillance of acute hepatitis.

## Author summary

We conducted a multicenter study of 1,721 adults with suspected acute hepatitis from five Brazilian regions. Anti-HEV IgG was detected in 5.2% of participants, with clear geographic and demographic variation, whereas anti-HAV IgG was much more frequent (78.6%). AHA was much more frequent than AHE: 87.1% vs. 12.9%. We also identified distinct epidemiological profiles: AHE affected older adults, was more frequent among women and heterosexual individuals, and occurred in both urban and rural settings. AHE cases had milder clinical and biochemical profiles than AHA cases, which showed higher aminotransferase levels and more frequent jaundice. These findings highlight an important gap in routine diagnostic testing for acute hepatitis in Brazil, because hepatitis E may be missed without targeted testing. Adding HEV testing to standard diagnostic panels could improve patient care and public health surveillance.

## Introduction

HEV was first recognized after large outbreaks of enterically transmitted non-A, non-B hepatitis in Asia, including epidemics in India and Afghanistan, and the viral agent was subsequently visualized as virus-like particles in stool samples [1]. HEV belongs to the family *Hepeviridae*, which includes multiple genera and species with human and animal hosts; among these, *Paslahepevirus balayani* includes the main human HEV genotypes, whereas *Rocahepevirus ratti* includes rat HEV strains with zoonotic potential [2]. HEV is a small, positive-sense single-stranded RNA virus with a genome of approximately 7.2 kb. The genome contains three principal open reading frames: ORF1 encodes the nonstructural polyprotein required for viral replication, ORF2 encodes the capsid protein, and ORF3 encodes a small multifunctional protein involved in virion release and pathogenesis. Virions are non-enveloped in feces but circulate in blood as quasi-enveloped particles surrounded by a host-derived lipid membrane [2].

HEV is now recognized as a major cause of acute viral hepatitis worldwide. The World Health Organization estimates that HEV caused approximately 20 million HEV infections worldwide every year, and 44,000 deaths in 2015 [3].

HEV genotypes differ in host range, geographic distribution, and transmission routes. Genotypes 1 and 2 infect humans and are mainly transmitted through fecally contaminated water, causing large outbreaks in regions with limited sanitation. Genotypes 3 and 4 circulate in animal reservoirs, particularly domestic pigs and wild boars, and cause sporadic zoonotic infections associated with raw or undercooked pork, game meat, shellfish, and other contaminated foods; transfusion-associated transmission has also been reported. Genotypes 5 and 6 have been detected in wild boars, whereas genotypes 7 and 8 occur in dromedaries and Bactrian camels, respectively, with rare human infection linked to camel-derived products [4–8].

HAV is another enterically transmitted hepatotropic virus and remains an important cause of acute viral hepatitis worldwide. HAV is a non-enveloped, positive-sense single-stranded RNA virus of the genus *Hepatovirus* in the family *Picornaviridae*. Three genotypes (I–III), each divided into subtypes A and B, infect humans; genotype I is the most prevalent worldwide, with subgenotype IA reported most frequently. HAV is transmitted mainly by the fecal–oral route through contaminated food or water or close person-to-person contact, including oral–anal sexual exposure. Unlike hepatitis B and C viruses, HAV does not cause chronic infection, but disease severity increases with age, and fulminant hepatitis may occur rarely [9,10].

The epidemiology of HAV has changed in many middle-income countries, including Brazil, where improvements in sanitation and the introduction of childhood vaccination have reduced early-life exposure while increasing the proportion of susceptible adolescents and adults in some settings. Universal HAV vaccination was incorporated into the Brazilian National Immunization Program in 2014 as a single-dose schedule for young children; however, heterogeneous coverage and regional socioeconomic differences may contribute to ongoing susceptibility and periodic outbreaks among older age groups and specific risk populations [11,12].

Because HAV and HEV share enteric routes of transmission and may present with overlapping clinical manifestations, distinguishing between these infections requires specific laboratory testing. In clinical practice, however, HEV is frequently not included in routine diagnostic algorithms for acute hepatitis in Brazil, which may lead to underdiagnosis, and misclassification of cases initially attributed to other causes, including HAV or drug-induced liver injury [5].

HEV epidemiology is commonly described according to two broad patterns. In endemic regions, particularly parts of Asia and Africa (especially in the Middle East), it is associated with large waterborne outbreaks linked to limited sanitation and unsafe drinking water [6]. In non-endemic or industrialized settings, sporadic autochthonous infections are increasingly recognized, frequently associated with zoonotic transmission, and can progress to chronic hepatitis in immunosuppressed individuals [13,14].

Risk factors for HEV infection and severe disease include residence in areas with inadequate sanitation, exposure to contaminated water, consumption of raw or undercooked pork or game meat, occupational contact with pigs, transfusion or invasive procedures, pregnancy, pre-existing liver disease, and immunosuppression, particularly in the context of genotype 3 infection [15].

During AHE, viremia usually peaks during incubation and early symptomatic phases, and serum HEV RNA may become undetectable within a few weeks after symptom onset, creating a narrow diagnostic window for PCR in blood. Viral RNA may be shed in feces for longer periods. Although most infections are self-limited, HEV can cause severe outcomes in vulnerable groups, including pregnant women, individuals with chronic liver disease, and immunosuppressed patients. Chronic infection, extrahepatic manifestations, and acute-on-chronic liver failure have also been described, particularly in association with genotype 3 infection [15–19].

In Brazil, available studies indicate heterogeneous HEV circulation across regions and populations. Reported anti-HEV IgG seroprevalence varies widely, from low levels in some northern populations to substantially higher estimates in the South and Southeast [20,21]. HEV has also been detected in several animal hosts and food-related sources, including pigs, wild rodents, horses, bovine liver, and capybaras, with genotype 3 detected where sequencing has been performed [20–27].

Despite evidence of zoonotic circulation, HEV testing is still not routinely performed in most Brazilian healthcare services. As a result, HEV infection may remain undetected among patients with acute hepatitis of unknown etiology, especially in those cases initially classified as HAV infection, drug-induced liver injury, or other causes. In countries where HEV testing has been incorporated into diagnostic workups, a proportion of presumed drug-induced liver injury cases has been reclassified as AHE [28].

This study aimed to (1) determine the frequency of AHE and evaluate the contribution of HEV infection to the burden of hepatitis among patients with suspected acute disease attended in public healthcare institutions in Brazil; (2) assess the prevalence of acquired immunity to HEV and HAV; and (3) compare demographic characteristics, risk factors, and clinical-laboratory findings associated with HEV and HAV infections.

These data are particularly relevant because HEV remains underdiagnosed in Brazil, and few multicenter studies have specifically evaluated AHE among patients with suspected acute hepatitis. Better characterization of HEV prevalence, exposure patterns, and clinical presentation may support improved diagnostic strategies, strengthen surveillance, and guide public health policies for acute hepatitis beyond hepatitis A, B, and C.

## Materials and methods

### Ethical statement

The project was approved by the Comissão Nacional de Ética em Pesquisa (CONEP; National Research Ethics Commission), the ethics committee responsible for approving multicenter studies such as this one, under the Certificado de Apresentação de Apreciação Ética (CAAE; Certificate of Presentation for Ethical Consideration) No. 00952818.4.1001.0071. All participants provided written informed consent before enrollment. For illiterate participants, consent was documented by fingerprint on the informed consent form. When participants were bedridden or unable to sign, consent was obtained from a close relative. The study protocol was conducted in accordance with the ethical principles of the Declaration of Helsinki.

### Study design and setting

#### Patients and samples.

This multicenter prospective observational study sequentially enrolled individuals aged ≥18 years who sought care for clinical signs or symptoms suggestive of acute liver disease between October 2019 and May 2023. Participants were recruited at 31 viral hepatitis referral services across 16 Brazilian states, representing the North, Northeast, South, Southeast, and Central West regions. A total of 1,721 samples were collected. All samples were validated according to the study protocol and had sufficient volume for testing HAV and HEV infection markers.

Participants were enrolled as part of the study “Study of the epidemiological and clinical characteristics of acute viral hepatitis in Brazilian healthcare services”, which has been previously described [29]. The protocol included biochemical tests, serological and molecular testing for hepatitis A, B, C, D, and E viruses and HIV, and additional laboratory investigations for arboviruses, herpesviruses, other viral infections, toxoplasmosis, leptospirosis, syphilis, and autoimmune hepatitis. Results concerning HAV have been published previously [30], whereas analyses of other etiologies are ongoing.

Eligibility criteria were defined as follows: (1) icteric symptomatic cases, defined as sudden onset of jaundice within six months before enrollment, with or without fever, malaise, nausea, vomiting, myalgia, acholic stools, or dark urine, and serum aminotransferase levels ≥1.5 times the upper limit of normal (ULN); (2) anicteric symptomatic non-jaundiced cases, defined as absence of jaundice with one or more symptoms suggestive of acute hepatitis and serum aminotransferase levels ≥1.5 times the ULN; and (3) anicteric asymptomatic cases, defined as absence of jaundice and symptoms but with a sudden increase in aminotransferase levels ≥1.5 times the ULN and/or a recognized close contact with a patient with confirmed viral hepatitis.

Blood samples collected by venipuncture were properly identified with sequential labels provided and identified with the patients’ full names and then centrifuged for serum or plasma separation. After collection, the samples were adequately processed, stored, and subsequently sent for analysis along with the completed documents to the Albert Einstein Clinical Pathology Laboratory in São Paulo, capital city of São Paulo State, Brazil.

A standardized questionnaire was used to collect demographic data, including sex at birth, gender identity, age, and geographic origin, as well as information on viral infections and individual risk factors.

#### Case definition of AHE.

AHE was defined using clinical and laboratory criteria informed by international and national recommendations. For this study, a case was classified as AHE when biochemical evidence of acute hepatitis, characterized by a recent elevation in serum aminotransferases, was accompanied by anti-HEV IgM reactivity and/or detectable HEV RNA by nucleic acid amplification testing. The international guidelines differ in the evidentiary weight assigned to these markers. EASL defines current acute HEV infection by detectable HEV RNA, with or without anti-HEV IgM, and does not classify isolated anti-HEV IgM positivity as current acute infection. In contrast, WHO surveillance guidance accepts anti-HEV IgM positivity in a patient with compatible acute hepatitis as laboratory confirmation, while noting that an additional marker, such as HEV RNA, increases diagnostic specificity. Thus, the operational case definition used in this study is consistent with the WHO surveillance approach but is broader than the EASL definition of current acute infection. Isolated anti-HEV IgG positivity was interpreted as evidence of previous exposure and was not used to define acute infection. Participants with anti-HEV IgG reactivity were analyzed separately to assess factors associated with previous HEV exposure [31,32].

#### Serology for HAV and HEV.

Qualitative detection of anti-HAV IgM and IgG antibodies and anti-HEV IgM and IgG antibodies was performed in human serum samples using commercial immunoassays. HAV serology was performed using ARCHITECT HAVAb-IgM and HAVAb-IgG assays based on chemiluminescent microparticle immunoassay technology (Abbott Diagnostics, Abbott Park, IL, USA). HEV serology was performed using the recomWell HEV IgM and IgG assays (Mikrogen Diagnostik, Neuried, Germany), a qualitative enzyme-linked immunosorbent assays based on recombinant antigens from HEV genotypes 1 and 3. All laboratory procedures and interpretation of results were performed according to the manufacturers’ instructions.

#### HAV and HEV RNA detection by reverse transcription–polymerase chain reaction (RT-PCR).

Viral RNA was extracted from all plasma samples before HAV and HEV RNA detection by real-time RT-PCR. Extraction was performed on the automated QIAsymphony platform using the QIAsymphony DSP Virus/Pathogen Mini Kit (Qiagen, Hilden, Germany), with input volumes of 350 µL for HAV testing and 650 µL for HEV testing, according to the manufacturer’s instructions. Extracted RNA was eluted in 60 µL, aliquoted, stored at −80°C, and thawed only once before amplification. HAV RNA was detected using the RealStar HAV RT-PCR Kit 1.0, and HEV RNA was detected using the RealStar HEV RT-PCR Kit 2.0 (Altona Diagnostics, Hamburg, Germany). Samples with cycle threshold (Ct) values ≤40 were considered positive.

#### Sequencing of HEV RNA.

HEV RNA-positive samples were subjected to molecular characterization by amplification and sequencing of partial fragments from two genomic regions using previously described primers: a 287-bp fragment of ORF1 [33] and a 348-bp fragment of ORF2 [34]. These fragments were amplified by nested PCR after reverse transcription. Reverse transcription and the first PCR round were performed using the SuperScript III One-Step RT-PCR System with Platinum Taq DNA Polymerase (Life Technologies, Foster City, CA, USA), and the second PCR round was performed using Platinum Taq DNA Polymerase (Life Technologies). Second-round PCR products were purified using the ExoSAP-IT PCR Clean-up Kit (GE Healthcare, Little Chalfont, Buckinghamshire, UK) and sequenced with the BigDye Terminator v3.1 Cycle Sequencing Kit (Applied Biosystems, Foster City, CA, USA), using the same primers as those used for nested PCR targeting the ORF1 and ORF2 regions. Dye-labeled products were analyzed on a 3500 Genetic Analyzer (Applied Biosystems). Sequence quality was assessed using Phred-Phrap, and consensus sequences were generated using CAP3 software.

### Statistical analysis

Statistical analyses were performed to compare demographic, behavioral, clinical, and laboratory variables across predefined groups. Two comparisons were evaluated: (1) patients with AHA versus patients with AHE, with acute infection defined by virus-specific IgM reactivity and/or viral RNA detection by RT-PCR; and (2) participants with evidence of previous HEV exposure, defined as anti-HEV IgG reactive, versus participants without evidence of previous HEV exposure, defined as anti-HEV IgG non-reactive.

Continuous variables were compared using Student’s t-test, and categorical variables were compared using the chi-squared test or Fisher’s exact test, as appropriate. Statistical significance was set at p < 0.05. Variables with p < 0.05 in bivariate analyses were included as candidate predictors in backward stepwise logistic regression models for the two predefined outcomes: AHA versus AHE and anti-HEV IgG reactive versus non-reactive status. Odds ratios (ORs) and 95% confidence intervals (95% CIs) were reported. Analyses were performed using RStudio statistical software (http://www.rstudio.com).

The dataset supporting the findings of this study has been deposited in the Open Science Framework (OSF) repository (https://osf.io/7gjsc).

## Results

### AHA and AHE cases

Among the 1,721 samples analyzed, 108 (6.3%) were classified as AHA: 99 were anti-HAV IgM reactive, of which 96 were also HAV RNA positive and three were HAV RNA negative, and nine were HAV RNA positive despite non-reactive anti-HAV IgM. Sixteen samples (0.93%) were classified as AHE; all were anti-HEV IgM reactive, and only one had detectable HEV RNA by RT-PCR. No simultaneous anti-HAV IgM and anti-HEV IgM reactivity was observed among the acute hepatitis cases. AHA was much more frequent than AHE, accounting for 87.1% of cases compared with 12.9% for AHE. Just the case with detectable HEV RNA was anti-CMV IgM positive. No case had detectable EBV VCA IgM. The median time from symptom onset to sampling was 12.5 days (mean: 15.75 days; IQR: 7.5-18.5 days; range: 0–47 days).

Among the 16 patients with AHE, the most frequently reported symptom was nausea, reported by 12 participants (75.0%). Anorexia and dark urine were each reported by nine participants (56.3%), while fever, jaundice, and vomiting were each reported by seven participants (43.8%). Acholic stools were reported by five participants (31.3%), hepatomegaly by four participants (25.0%), and splenomegaly and rash by two participants each (12.5%). These cases were added between 0–37 days after symptom onset. Only symptomatic patients were diagnosed as AHE.

Demographic and laboratory analyses revealed significant differences between the acute hepatitis groups. The mean age was higher among patients with AHE than among those with AHA (49.4 versus 34.9 years; p = 0.004). Mean ALT and AST levels were significantly higher in the AHA group (ALT: 1,150 U/L; AST: 599 U/L) than in the AHE group (ALT: 233 U/L; AST: 113 U/L; p < 0.001). The ALT/AST ratio and total and direct bilirubin levels were also higher in the AHA group (p = 0.034 and p = 0.036, respectively).

Demographic, clinical, and behavioral characteristics of patients with AHA and AHE are shown in **Table 1**; only statistically significant associations are presented, whereas non-significant results are provided in **S1 Table**. AHA was more frequent among males, participants from the Southeast region, individuals living in urban areas, bisexual or homosexual participants, cis men, symptomatic jaundiced cases, men who have sex with men (MSM), and individuals with non-reactive anti-*Toxoplasma gondii* IgG. In contrast, AHE was more frequent among females, participants from the South region, individuals living in rural areas, heterosexual participants, cis women, symptomatic non-jaundiced cases, participants with previous surgery, non-MSM, and individuals with reactive anti-*Toxoplasma gondii* IgG.

**Table 1:** Demographic, clinical, and behavioral characteristics of patients with acute hepatitis A (AHA) and acute hepatitis E (AHE).

	Total n = 124	AHA n = 108	AHE n = 16	p value *
***Sex at birth***				***< 0.001***
Female	32	22 (20.4%) %	10 (62.5 %)	
Male	92	86 (79.6 %)	6 (37.5 %)	
***Region of residence***				***0.036***
Central West	2	1 (0.9 %)	1 (6.3 %)	
North	2	1 (0.9 %)	1 (6.3 %)	
Northeast	2	1 (0.9 %)	1 (6.3 %)	
South	45	38 (35.2 %)	7 (43.8 %)	
Southeast	73	67 (62.0 %)	6 (37.5 %)	
***Geographical characteristics of the place of residence***				***0.016***
Rural area	2	0 (0.0 %)	2 (12.5 %)	
Urban area	122	108 (100.0 %)	14 (87.5 %)	
***Sexual orientation***				***0.004***
Bisexual	8	8 (8.3 %)	0 (0.0 %)	
Heterosexual	72	56 (58.3 %)	16 (100.0 %)	
Homosexual	32	32 (33.3 %)	0 (0.0 %)	
***Gender identity***				***0.017***
Cis man	81	75 (75.8 %)	6 (40.0 %)	
Cis woman	31	22 (22.2 %)	9 (60.0 %)	
Trans woman	1	1 (1.0 %)	0 (0.0 %)	
Nonbinary	1	1 (1.0 %)	0 (0.0 %)	
***Inclusion Criteria***				***< 0.001***
Anicteric asymptomatic	4	4 (3.7%)	0 (0.0 %)	
Anicteric symptomatic	20	11 (10.2 %)	9 (56.2 %)	
Icteric symptomatic	100	93 (86.1 %)	7 (43.8 %)	
***Jaundice***				***p < 0.001***
No	24	15 (13.9 %)	9 (56.3 %)	
Yes	100	93 (86.1 %)	7 (43.8 %)	
***Choluria***				***0.019***
No	25	18 (16.7 %)	7 (43.8 %)	
Yes	99	90 (83.3 %)	9 (56.3 %)	
***Surgery***				***0.005***
No	92	85 (78.7 %)	7 (43.8 %)	
Yes	32	23 (21.3 %)	9 (56.3 %)	
***Men who have sex with men (MSM)***				***0.003***
MSM	38	38 (35.2 %)	0 (0.0 %)	
Non MSM	86	70 (64.8 %)	16 (100.0 %)	
**Toxoplasmosis Anti-IgG**				***< 0.001***
Indeterminate	1	1 (0.9 %)	0 (0.0 %)	
Non-reactive	87	82 (75.9 %)	5 (31.3 %)	
Reactive	36	25 (23.1 %)	11 (68.8 %)	

**Note:** Bold values are statistically significant. *p value corresponding to comparisons between patients with AHA and AHE

### Demographic, clinical and laboratorial data of the HEV RNA positive case

The patient was a 32-year-old cisgender man residing in an urban area in Rondônia State, North Brazil. He identified as heterosexual, did not belong to the MSM group, and reported two sexual partners during the previous six months. He had a history of prior surgery (type not specified). The plasma sample was collected 19 days after symptom onset. At enrollment, he reported nausea, fever, and anorexia, without choluria or clinical jaundice, and was classified as anicteric. He reported no seafood consumption. The patient denied recent domestic or international travel.

Biochemical liver tests showed elevated aminotransferases and gamma-glutamyl transferase: AST 224 U/L, ALT 259 U/L, and GGT 259 U/L. Alkaline phosphatase was 99 U/L. Total and fractionated bilirubin levels were normal (total, 0.6 mg/dL; direct, 0.2 mg/dL; indirect, 0.4 mg/dL), consistent with the absence of jaundice. Total IgG was not available.

Laboratory evaluation demonstrated reactive anti-HEV IgM and detectable HEV RNA in plasma by qualitative RT-qPCR (Ct 38.1). Attempts to amplify the target regions for sequencing and genotyping were unsuccessful. HBsAg, total anti-HBc, and anti-HCV were non-reactive. Anti-HIV serology was reactive, with an HIV RNA level >5,400,000 IU/mL; molecular characterization identified HIV-1 group M, subtype B. Anti-HAV IgG was reactive, and HAV RNA was not detected, consistent with previous HAV exposure. *Leptospira* serology was non-reactive, and toxoplasmosis serology was reactive. Autoimmune testing showed antinuclear antibody reactivity with a fine-speckled nuclear pattern at a titer of 1:80 and a speckled cytoplasmic pattern at 1:80. Nucleolar and mitotic-apparatus patterns were non-reactive. Anti-LKM and ASMA-2 patterns involving the muscularis mucosa, glomerular mesangium, vascular smooth-muscle sheath, and intercellular tubular fibrils were non-reactive. Anti-actin IgG was 38.6 U; however, total IgG was unavailable, and these findings were reported descriptively without establishing autoimmune hepatitis. The concomitant HIV infection may have contributed to nonspecific autoantibody reactivity through immune dysregulation but not to the detection of HEV RNA by RT-qPCR.

### Anti-HAV and Anti-HEV IgG cases

Anti-HAV IgG reactivity was detected in 1,352 participants (78.6%). Evidence of previous HEV exposure, defined by anti-HEV IgG reactivity, was identified in 89 participants (5.2%).

**Table 2** presents the demographic, clinical, and behavioral characteristics associated with anti-HEV IgG reactivity; only statistically significant associations are shown, whereas non-significant results are provided in **S2 Table**. Anti-HEV IgG was more frequent among residents of the South, Southeast, and Central West regions and increased with age, from 1.0%–1.7% among individuals aged 18–34 years to 13.0% among those aged ≥65 years. Other associated factors included heterosexual orientation, absence of AHA, previous leptospirosis, reactive anti-HAV IgG, reactive total anti-HBc, and reactive anti-Toxoplasma gondii IgG. Anti-HEV IgG seropositivity was lower among MSM than among non-MSM individuals (1.4% versus 5.7%).

**Table 2:** Demographic, clinical, and behavioral characteristics associated with anti-HEV IgG reactivity.

	n = 1720	IgG (−) n = 1631	IgG (+) n = 89	*p* value^a^
***Region of residence***				**0.007**
Central West	125	118 (94.4 %)	7 (5.6 %)	
North	244	239 (98.0 %)	5 (2.0 %)	
Northeast	212	207 (97.6 %)	5 (2.4 %)	
South	385	356 (92.5 %)	29 (7.5 %)	
Southeast	754	711 (94.3 %)	43 (5.7 %)	
***Age group***				**< 0.001**
18-24	181	178 (98.3 %)	3 (1.7 %)	
25-29	207	205 (99.0 %)	2 (1.0 %)	
30-34	210	207 (98.6 %)	3 (1.4 %)	
35-39	209	200 (95.7 %)	9 (4.3 %)	
40-44	212	193 (91.0 %)	19 (9.0 %)	
45-49	163	154 (94.5 %)	9 (5.5 %)	
50-54	152	143 (94.1 %)	9 (5.9 %)	
55-59	119	111 (93.3 %)	8 (6.7 %)	
60-64	106	100 (94.3 %)	6 (5.7 %)	
65+	161	140 (87.0 %)	21 (13.0 %)	
***Sexual orientation***				**0.008**
Bisexual	26	26 (100.0 %)	0 (0.0 %)	
Heterosexual	1396	1316 (94.3 %)	80 (5.7 %)	
Homosexual	215	212 (98.6 %)	3 (1.4 %)	
***AHA***				**0.040**
Absent	1612	1524 (94.5 %)	88 (5.5 %)	
Present	108	107 (99.1 %)	1 (0.9 %)	
***Leptospirosis***				**0.002**
Non-reactive	1701	1617 (95.1 %)	84 (4.9 %)	
Reactive	19	14 (73.7 %)	5 (26.3 %)	
***Seafood***				**0.002**
No	1351	1270 (94.0 %)	81 (6.0 %)	
Yes	367	359 (97.8 %)	8 (2.2 %)	
***Anti HAV IgG***				**< 0.001**
Non-reactive	368	365 (99.2 %)	3 (0.8 %)	
Reactive	1349	1263 (93.6 %)	86 (6.4 %)	
***Anti-HBc Total***				**0.012**
Non-reactive	1395	1332 (95.5 %)	63 (4.5 %)	
Reactive	321	295 (91.9 %)	26 (8.1 %)	
***IgG Anti-Toxoplasmosis***				**0.0003**
Non-reactive	787	763 (96.9 %)	24 (3.1 %)	
Reactive	924	860 (93.1 %)	64 (6.9 %)	
***MSM***				**0.005**
MSM	218	215 (98.6 %)	3 (1.4 %)	
Non MSM	1502	1416 (94.3 %)	86 (5.7 %)	

**Note:** Bold values are statistically significant. *p value corresponding to comparisons between anti-HEV IgG (-) and anti-HEV IgG (+) patients

**Table 3** presents the logistic regression analysis of factors associated with anti-HEV IgG seropositivity. Individuals from the North (OR: 0.19; 95% CI: 0.06–0.49; p = 0.001) and Northeast (OR: 0.20; 95% CI: 0.06–0.53; p = 0.003) regions had lower odds of anti-HEV IgG seropositivity than those from the South, Southeast, and Central West regions. No age group showed a statistically significant association in the multivariable model, although individuals aged 40–44 years showed a trend toward higher odds compared with those aged 18–24 years (OR: 3.07; 95% CI: 0.96–13.7; p = 0.087). Non-heterosexual orientation showed a trend toward lower odds of anti-HEV IgG seropositivity (OR: 0.31; 95% CI: 0.07–0.90; p = 0.060), although this association did not reach statistical significance. Reactive anti-HAV IgG was strongly associated with increased odds of anti-HEV IgG seropositivity (OR: 5.77; 95% CI: 2.00–24.5; p = 0.005).

**Table 3:** Logistic regression analysis for factors associated with anti‐HEV IgG seropositivity.

	OR^a^	95% CI^a^	*p* value
***Region of residence***			
Central West	0.73	0.28–1.69	0.5
North	0.19	0.06–0.49	**0.001**
Northeast	0.20	0.06–0.53	**0.003**
South	—	—	
Southeast	0.75	0.44–1.28	0.3
***Age group***			
18-24	—	—	
25-29	0.48	0.06–3.02	0.4
30-34	0.44	0.06–2.73	0.4
35-39	1.59	0.44–7.50	0.5
40-44	3.07	0.96–13.7	0.087
45-49	1.07	0.28–5.21	>0.9
50-54	1.35	0.36–6.47	0.7
55-59	1.30	0.34–6.41	0.7
60-64	1.12	0.27–5.65	0.9
65+	2.50	0.77–11.4	0.2
***Sexual orientation***			
Heterosexual	—	—	
No Heterosexual	0.31	0.07–0.90	0.060
***AHA***			
Non-reactive	—	—	
Reactive	0.23	0.01–1.14	0.2
***Leptospirose***			
Non-reactive	—	—	
Reactive	3.00	0.63–10.8	0.12
***Seafood***			
No	—	—	
Yes	0.52	0.21–1.11	0.12
***IgG Anti HAV***			
Non-reactive	—	—	
Reactive	5.77	2.00–24.5	**0.005**

**Note:** Bold values are statistically significant.

^**a**^ **CI  =**  confidence interval, OR  =  odds ratio.

Anti-HEV IgG seroprevalence varied across age groups and increased progressively with age (Fig 1). Frequencies were low among younger participants aged 18–34 years, ranging from 1.0% to 1.7%. Seroprevalence rose to 4.3% at ages 35–39 years and 9.0% at ages 40–44 years. It then fluctuated between 5.5% and 6.7% from ages 45–64 years. The highest prevalence was observed among participants aged ≥65 years (13.0%), supporting a positive association between older age and previous HEV exposure.

**Fig 1:**
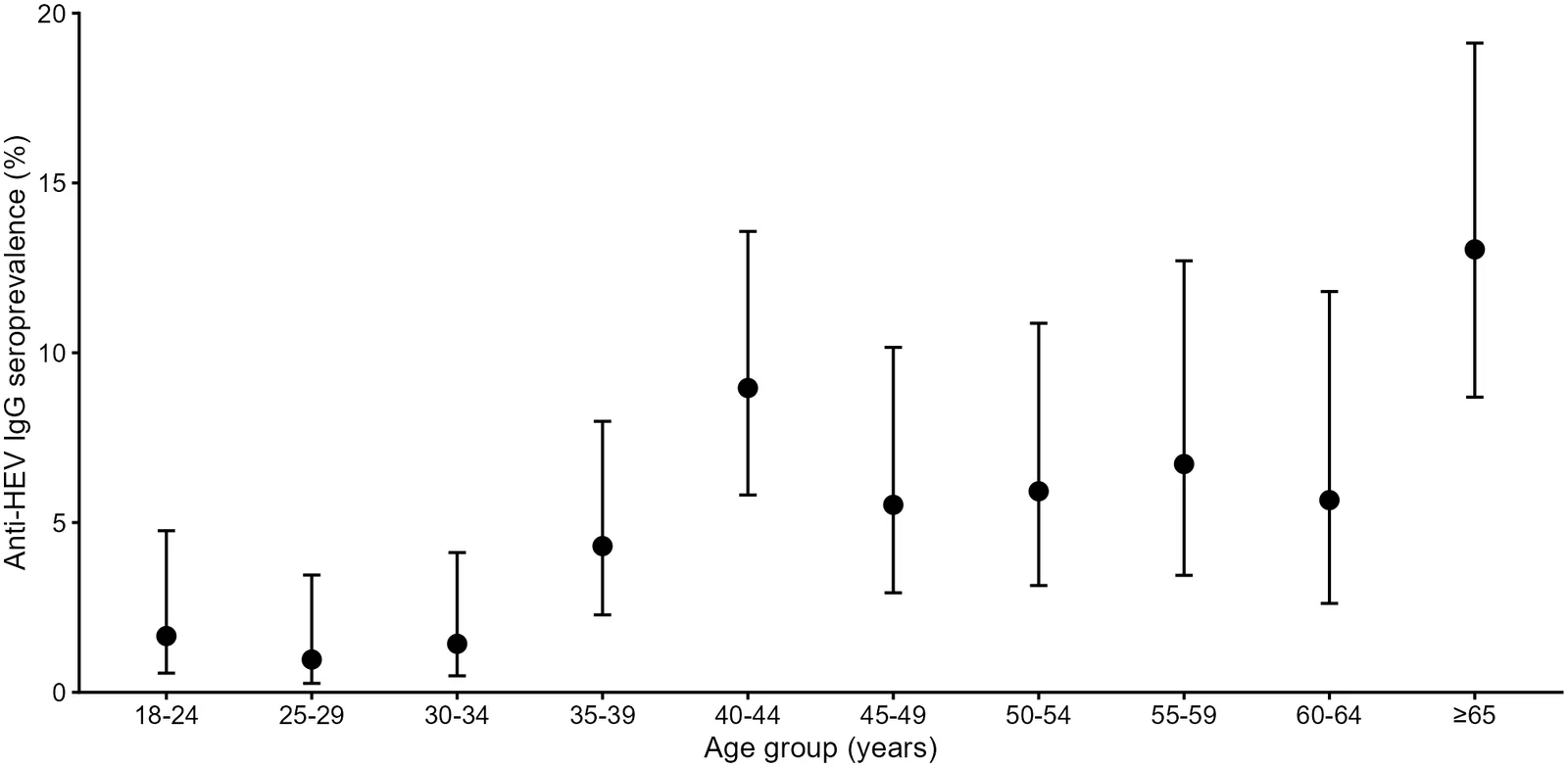
Anti‐HEV IgG seroprevalence by age group.

The final figure in the Results section summarizes the regional distribution of AHE cases and anti-HEV IgG seropositivity across Brazil (Fig 2).

**Fig 2:**
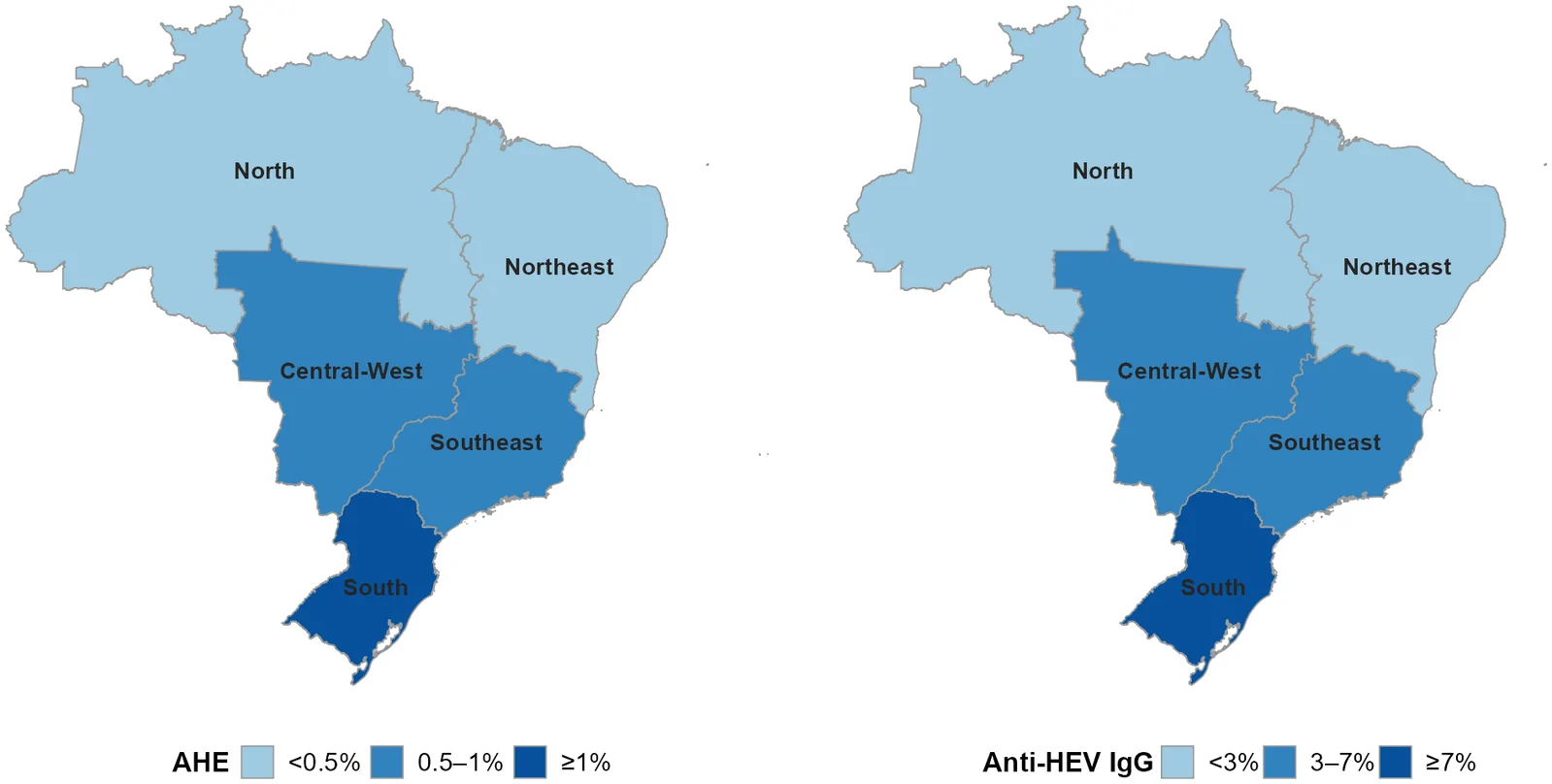
Regional distribution of acute hepatitis E cases and anti-HEV IgG seropositivity in Brazil. (A) Percentage distribution of the 16 acute hepatitis E (AHE) cases across the five Brazilian macro-regions. (B) Anti-HEV IgG seroprevalence within each Brazilian macro-region among the study participants.

The percentages in panel A represent the regional distribution of AHE cases, whereas those in panel B represent the proportion of participants who were anti-HEV IgG positive within each region. Fig 2 is original and was authored by the manuscript's authors. The map was generated in R from regional boundary shapefiles obtained through the geobr R package (version 2.0.0), which provides official geographic data from the Brazilian Institute of Geography and Statistics (IBGE: https://www.ibge.gov.br/en/home-eng.html?lang=en-GB).

## Discussion

A multicenter, prospective, observational study was conducted between October 2019 and May 2023, including 1,721 patients (≥18 years) with suspected acute hepatitis from 31 referral centers across 16 Brazilian states. This study included referral centers from all five Brazilian macro-regions. Brazil is continental country; its extension is 48% of the land area and 50% of the population of South America. Brazil exhibits substantial demographic, geographic, socioeconomic, environmental, and healthcare-access heterogeneity across its five macroregions (North, Northeast, Central-West, Southeast, and South), which may influence the epidemiology of HAV and HEV infections. These regions differ markedly in population density, urbanization patterns, sanitation coverage, water supply infrastructure, income levels, livestock production systems, environmental conditions, and access to healthcare services. The North and Northeast regions generally present lower sanitation coverage and greater social vulnerability, whereas the South and Southeast are characterized by higher urbanization, better healthcare access, and more intensive swine production, a recognized reservoir of zoonotic HEV genotype 3. Such regional disparities may affect both the risk of exposure to HEV through environmental and zoonotic transmission routes and the likelihood of detection and diagnosis*.*

Importantly, the present study included only individuals aged 18 years or older. This characteristic may explain the higher frequency of HAV and HEV cases observed in the South and Southeast regions. In areas with better sanitation and lower exposure to enteric pathogens during childhood, a larger proportion of the population may remain susceptible into adulthood, resulting in a higher likelihood of infection being detected among adults. Conversely, in regions with poorer sanitation, exposure may occur earlier in life, leading to a lower proportion of susceptible adults. Therefore, regional differences in hepatitis occurrence may reflect not only variations in viral circulation but also disparities in sanitation, age-related exposure patterns, and healthcare access.

A total of 124 acute infections were identified; 12.9% were identified as AHE and 87.1% as AHA. A review on acute viral hepatitis around the world based on the Global Burden of Disease study 2021 estimated the following number of cases: AHA (160.86 million) and AHE (19.37 million). Projections suggest that by 2030, the global number of AHA cases could decrease to 152.9 million while the global number of AHE cases will rise to 20.5 million [35]. The AHA/AHE proportion we found in Brazil was 6.77 while in the world in 2021 it was 8.30. Other studies addressing AHE should be carried out in our country to further confirm the data shown herein and to better understand the burden of this infection in Brazil. The limited detection of HEV RNA among patients classified as AHE may partly reflect the interval between symptom onset and sample collection (median, 12.5 days; mean, 15.7 days), given the relatively short duration of viremia in acute HEV infection. By the time blood was collected, circulating viral RNA may already have declined below the assay detection limit in some patients. Future studies should consider collecting stool specimens in addition to blood, because fecal HEV RNA excretion may persist longer and may extend the window for molecular detection.

In our nationwide study using both serologic and molecular methods, anti‑HAV IgG was detected in 1,352 individuals (78.5%) vs. anti-‑HEV IgG in 89 (5.17%). This large gap likely reflects true epidemiologic differences: HAV is widespread in Brazil with high cumulative immunity from past infection and vaccination, while HEV transmission appears more focal, less common, and underrecognized due to limited surveillance and variable diagnostic availability. These findings support strengthening HEV testing and targeted epidemiologic studies.

Individuals from the North and Northeast exhibited significantly lower odds of anti-HEV IgG seropositivity compared to those from the South, Southeast and Central West regions. Previous data showed considerable heterogeneity across the regions and within distinct populations in the same geographic area. For example, prior studies in the North region reported varied seroprevalence rates: 4.5% in urban and 12.9%in rural areas [36]. The South region consistently reported the highest seroprevalence rates, often exceeding 10%, with some studies reaching up to 65% [37]. In contrast, studies in the Southeast, although variable, typically give results around 11% [38].

To address the geographic context of the sampled population, it is important to emphasize that the study included referral centers distributed across all five Brazilian macro-regions, encompassing heterogeneous demographic, socioeconomic, and environmental settings. These differences may help explain the regional variation observed in HEV exposure. The higher anti-HEV IgG seroprevalence in the South, Southeast, and Central West may reflect distinct patterns of urbanization, food consumption, animal exposure, livestock production, and access to diagnostic services, whereas the lower frequency observed in the North and Northeast may be influenced by differences in the sampled populations, healthcare-seeking patterns, and local transmission dynamics. In addition, the occurrence of AHE cases in both urban and rural settings suggests that HEV exposure in Brazil is not restricted to a single epidemiological scenario and may involve multiple routes of transmission, including environmental and zoonotic pathways. However, because the study was conducted in referral healthcare services and not as a population-based survey, regional comparisons should be interpreted with caution.

Our findings align with prior evidence showing substantial regional heterogeneity in HEV circulation in Brazil, with higher seroprevalence in the South, Southeast, and Central West and markedly lower levels in the North and Northeast. This pattern mirrors the epidemiological landscape described in previous studies and reinforces that HEV transmission in Brazil is focal and unevenly distributed. By providing standardized, multicenter data, our study confirms these regional disparities and demonstrates that HEV accounts for a measurable proportion of enterically transmitted acute hepatitis, supporting the need for routine HEV testing in the diagnostic evaluation of acute hepatitis.

A progressive increase in anti-HEV IgG seroprevalence was noted with advancing age, peaking at 13.0% among individuals aged ≥65 years. Younger age groups (18–34 years) had low seroprevalence (1.0–1.7%), with an increase beginning from the 35–39 age group. Although logistic regression did not reveal statistically significant associations for all age categories, a trend toward increased odds of seropositivity was observed in the 40–44 age group (OR: 3.07, 95% CI: 0.96–13.7; p = 0.087), compared to the 18–24 age group. These findings align with previous data from Brazil and other countries, where HEV seroprevalence generally increases with age [4].

Notable demographic and clinical differences were identified between AHA and AHE infections. Patients with AHE had a higher mean age (49.4 years) compared to those with AHA (34.9 years; p = 0.004). AHA was significantly associated with male sex (79.6%), whereas AHE was more prevalent among females (62.5%). AHA was also associated with bisexual (8.3%) and homosexual (33.3%) orientation, while all AHE cases occurred in individuals identifying as heterosexual (100%). These results support the established link between HAV and outbreaks among MSM, especially in high-income settings [30]. Indeed, this study confirmed a significant association between AHA and MSM (35.2% of HAV cases), whereas no AHE was reported in this group. More studies should be carried out and despite conflicting data, for the moment, MSM with unexplained jaundice, transaminitis, renal disease or neurological disease should be tested for HEV [39].

All AHA cases occurred in urban areas (100.0%), whereas AHE cases were reported in both urban and rural settings (12.5% rural). Clinically, AHA was associated with icteric symptomatic presentation (86.1%), with smaller proportions of anicteric symptomatic (10.2%) and anicteric asymptomatic cases (3.7%). In contrast, all AHE cases were symptomatic, with 56.3% presenting without jaundice and 43.8% with jaundice. These findings differ from existing literature, which typically reports jaundice in 5%–30% of HEV cases [40]. Although jaundice and choluria were less frequent in AHE vs. AHA (43.8% vs. 86.1% and 56.3% vs. 83.3%, respectively), the clinical presentations were similar, reinforcing the diagnostic challenges and the need to detect specific etiological markers for an accurate diagnosis. Anti-HEV IgM positivity has been detected in asymptomatic individuals in some previous population-based studies supporting the notion of subclinical circulation of the virus and highlighting the potential underestimation of true HEV seroprevalence [37,41]. It is noteworthy that in this study all patients that were diagnosed as AHE were symptomatic.

The markedly higher aminotransferase levels in the AHA group (ALT 1,150 U/L; AST 599 U/L) compared with the AHE group (ALT 233 U/L; AST 113 U/L) are in keeping with the typical biochemical profile of AHA, which often causes abrupt and pronounced hepatocellular injury with very large transaminase elevations. This difference likely reflects distinct pathophysiological and clinical patterns: HAV usually produces a sudden, symptomatic hepatic illness with high peak ALT and AST, whereas HEV—particularly sporadic HEV‑3 infections in older adults—tends to show more variable enzyme responses and a wider clinical spectrum that can include subclinical or milder biochemical abnormalities [42]. Differences in timing of sampling relative to symptom onset, as well as case characteristics such as age, comorbidities, and immune status, may further influence the observed group disparities.

The higher ALT/AST ratio observed in the AHA group indicates a disproportionately greater ALT rise relative to AST, consistent with a predominantly hepatocellular pattern of injury in acute HAV infection. The concurrent elevations in total and direct bilirubin suggest more pronounced impairment of bilirubin conjugation and/or excretion in HAV cases, producing more evident cholestasis and clinical jaundice than seen in the HEV group [43].

We found a significant association between acute HEV infection and anti-*Toxoplasma* IgG positivity (68.8% of HEV cases), which may reflect a common route of exposure, particularly ingestion of raw or undercooked meat. Previous studies reported an association between HEV and *Toxoplasma* markers in meat juice samples from pigs [44,45], but further research is needed to confirm this relationship in humans—particularly in Brazil after the large toxoplasmosis outbreak in Santa Maria, Rio Grande do Sul [46].

Interestingly, reactive anti-HEV IgG was strongly associated with reactive anti-HAV IgG, anti-HBc, and leptospirosis, indicating prior immunity or past exposure to hepatitis A and B, respectively, together with a superimposed leptospiral infection occurring alongside HEV; this pattern supports ongoing, overlapping zoonotic or environmental exposures in patients with a concurrent risk for previous hepatitis A or B infections [47,48].

Importantly, we did not assess HAV vaccination status, which limits the epidemiological interpretation of anti-HAV IgG positivity. Anti-HAV IgG indicates immunity but does not distinguish between vaccine-induced immunity and previous natural infection, whereas anti-HAV IgM is indicative of acute or recent infection [49]. Several experimental or research-based approaches have been proposed to address this limitation, including IgG avidity assays and antibody responses against non-structural HAV proteins [50–52]. However, these methods have important constraints, such as transient responses, limited diagnostic performance, and lack of routine clinical availability. Thus, in most clinical and epidemiological settings, accurate discrimination between vaccination and natural infection relies primarily on documented vaccination history rather than laboratory markers.

A recent scoping review of viral hepatitis in Brazil highlighted the substantial heterogeneity of HEV seroprevalence estimates reported across the country, with values ranging from 0.9% to 59.4% depending on the region and population studied. The review also demonstrated that most available evidence originates from regional studies conducted in selected populations, including blood donors, transplant recipients, people who use drugs, rural communities, and individuals with chronic liver disease, limiting the direct comparability of findings across settings. Furthermore, differences in study design, population characteristics, sampling strategies, geographic coverage, and laboratory methodologies may all contribute to the variability observed in the literature. In relation to acute hepatitis E, the review noted that confirmed cases remain uncommon and are likely underdetected, with most reports describing sporadic acute infections identified incidentally during investigations of unexplained hepatitis [53].

In line with these observations, the interpretation of HEV seroprevalence estimates should be approached with caution, as reported differences may reflect not only true epidemiological variation but also methodological heterogeneity among studies [54–56]. In addition, changes in anti-HEV serological assays over time may further influence comparisons between studies. In the present study, the use of a single standardized serological assay and harmonized procedures across all participating centers enhanced the internal consistency of the analyses and allowed direct comparisons among regions and population groups, providing robust nationwide data on HEV epidemiology in Brazil.

## Conclusions

These findings should be interpreted in light of some limitations, including the referral-center-based design, which may not fully represent the general Brazilian population; the relatively small number of confirmed AHE cases, which limited subgroup analyses and reduced the statistical power to explore associations with specific environmental and dietary exposures collected in the questionnaire; the absence of complete information on HAV vaccination status; and the possibility that HEV RNA detection was affected by the short viremic window and timing of sample collection. On the other hand, the strength of this paper is that it provides more comparable seroprevalence estimates across different regions, as the same assay was consistently used for all analyzed samples.

The findings confirm significant regional and demographic differences in HEV seroprevalence, with higher rates in the South and Southeast, and a progressive increase with advancing age. Importantly, HEV accounted for 12.9% of enterically transmitted acute viral hepatitis cases, reinforcing its relevance as a public health concern. The observed epidemiological contrasts between HAV and HEV, in terms of age, sex, sexual orientation, and urban/rural distribution, underscore distinct transmission dynamics that require differentiated prevention strategies. Nevertheless, the frequent occurrence of common hepatitis symptoms among HEV cases emphasizes the need for routine inclusion of HEV testing in the diagnostic evaluation of acute hepatitis of unknown etiology. Collectively, these results call for greater awareness, improved surveillance, and the development of targeted public health policies aimed at reducing the burden of both HAV and HEV in Brazil.

## Supporting information

S1 Table Demographic, clinical, and behavioral characteristics of patients with acute hepatitis A (AHA) and acute hepatitis E (AHE). (DOCX)

S2 Table Demographic, clinical, and behavioral characteristics associated with anti-HEV IgG reactivity. (DOCX)
