# Supplementary material for: Hepatitis E in suspected acute hepatitis cases in Brazil: A comparison with hepatitis A
Source: PLoS Negl Trop Dis. 2026 Oct 26;20(10):e0014834. doi: 10.1371/journal.pntd.0014834 (PMC13766607; doi:10.1371/journal.pntd.0014834)
Supplement: Supplementary file 1 [file pntd.0014834.s001.docx]

S1 Table. Demographic, clinical, and behavioral characteristics of patients with acute hepatitis A (AHA) and acute hepatitis E (AHE)

| **Characteristic** | **Overall, N = 124^1^** | **AHA, N = 108^1^** | **AHE, N = 16^1^** | **p-value^2^** |
| --- | --- | --- | --- | --- |
| **Demographic data** | | | | |
| **Age (years)** | 34.0 (28.5–41.5) | 34.0 (27.5–40.0) | 45.0 (34.0–63.0) | 0.001 |
| **Sex at birth** |  |  |  | <0.001 |
| Female | 32 (25.8%) | 22 (20.4%) | 10 (62.5%) |  |
| Male | 92 (74.2%) | 86 (79.6%) | 6 (37.5%) |  |
| **Gender identity** |  |  |  | 0.017 |
| Cis man | 81 (71.1%) | 75 (75.8%) | 6 (40.0%) |  |
| Cis woman | 31 (27.2%) | 22 (22.2%) | 9 (60.0%) |  |
| Trans woman | 1 (0.9%) | 1 (1.0%) | 0 (0.0%) |  |
| Nonbinary | 1 (0.9%) | 1 (1.0%) | 0 (0.0%) |  |
| **Race** |  |  |  | 0.536 |
| White | 60 (63.8%) | 55 (65.5%) | 5 (50.0%) |  |
| Black | 13 (13.8%) | 11 (13.1%) | 2 (20.0%) |  |
| Mixed | 20 (21.3%) | 17 (20.2%) | 3 (30.0%) |  |
| Asian | 1 (1.1%) | 1 (1.2%) | 0 (0.0%) |  |
| **Geographical characteristics of the place of residence** |  |  |  | 0.016 |
| Urban area | 122 (98.4%) | 108 (100.0%) | 14 (87.5%) |  |
| Rural area | 2 (1.6%) | 0 (0.0%) | 2 (12.5%) |  |
| **Region of residence** |  |  |  | 0.036 |
| North | 2 (1.6%) | 1 (0.9%) | 1 (6.3%) |  |
| Northeast | 2 (1.6%) | 1 (0.9%) | 1 (6.3%) |  |
| Central-West | 2 (1.6%) | 1 (0.9%) | 1 (6.3%) |  |
| Southeast | 73 (58.9%) | 67 (62.0%) | 6 (37.5%) |  |
| South | 45 (36.3%) | 38 (35.2%) | 7 (43.8%) |  |
| **Marital status** |  |  |  | 0.007 |
| Single | 73 (59.8%) | 65 (61.3%) | 8 (50.0%) |  |
| Married | 28 (23.0%) | 24 (22.6%) | 4 (25.0%) |  |
| Stable union | 14 (11.5%) | 14 (13.2%) | 0 (0.0%) |  |
| Separated | 2 (1.6%) | 0 (0.0%) | 2 (12.5%) |  |
| Divorced | 4 (3.3%) | 2 (1.9%) | 2 (12.5%) |  |
| Widowed | 1 (0.8%) | 1 (0.9%) | 0 (0.0%) |  |
| **Inclusion** **criteria** | | | | |
| **Inclusion criteria** |  |  |  | <0.001 |
| Anicteric asymptomatic | 4 (3.2%) | 4 (3.7%) | 0 (0.0%) |  |
| Anicteric symptomatic | 20 (16.1%) | 11 (10.2%) | 9 (56.3%) |  |
| Icteric symptomatic | 100 (80.6%) | 93 (86.1%) | 7 (43.8%) |  |
| **Clinical data** | | | | |
| **Jaundice** |  |  |  | <0.001 |
| No | 24 (19.4%) | 15 (13.9%) | 9 (56.3%) |  |
| Yes | 100 (80.6%) | 93 (86.1%) | 7 (43.8%) |  |
| **Fever** |  |  |  | 0.182 |
| No | 50 (40.3%) | 41 (38.0%) | 9 (56.3%) |  |
| Yes | 74 (59.7%) | 67 (62.0%) | 7 (43.8%) |  |
| **Nausea** |  |  |  | 0.757 |
| No | 28 (22.6%) | 24 (22.2%) | 4 (25.0%) |  |
| Yes | 96 (77.4%) | 84 (77.8%) | 12 (75.0%) |  |
| **Vomiting** |  |  |  | >0.999 |
| No | 67 (54.0%) | 58 (53.7%) | 9 (56.3%) |  |
| Yes | 57 (46.0%) | 50 (46.3%) | 7 (43.8%) |  |
| **Choluria** |  |  |  | 0.019 |
| No | 25 (20.2%) | 18 (16.7%) | 7 (43.8%) |  |
| Yes | 99 (79.8%) | 90 (83.3%) | 9 (56.3%) |  |
| **Anorexia** |  |  |  | 0.148 |
| No | 35 (28.2%) | 28 (25.9%) | 7 (43.8%) |  |
| Yes | 89 (71.8%) | 80 (74.1%) | 9 (56.3%) |  |
| **Pale stools** |  |  |  | 0.067 |
| No | 58 (46.8%) | 47 (43.5%) | 11 (68.8%) |  |
| Yes | 66 (53.2%) | 61 (56.5%) | 5 (31.3%) |  |
| **Hepatomegaly** |  |  |  | >0.999 |
| No | 93 (75.0%) | 81 (75.0%) | 12 (75.0%) |  |
| Yes | 31 (25.0%) | 27 (25.0%) | 4 (25.0%) |  |
| **Splenomegaly** |  |  |  | >0.999 |
| No | 107 (86.3%) | 93 (86.1%) | 14 (87.5%) |  |
| Yes | 17 (13.7%) | 15 (13.9%) | 2 (12.5%) |  |
| **Biochemical laboratory data** | | | | |
| **ALT (U/L)** | 495.0 (121.0–1,528.5) | 570.0 (130.5–1,684.0) | 114.0 (56.0–468.5) | 0.001 |
| **AST (U/L)** | 164.5 (60.0–439.5) | 171.0 (69.5–697.0) | 84.0 (43.0–180.0) | 0.012 |
| **GGT (U/L)** | 215.0 (95.0–365.0) | 210.0 (90.5–362.5) | 228.0 (119.5–563.0) | 0.350 |
| **Alkaline phosphatase (U/L)** | 171.5 (120.0–233.5) | 173.5 (126.5–220.0) | 150.5 (104.0–362.5) | 0.502 |
| **Total bilirubin (mg/dL)** | 3.2 (1.0–7.1) | 4.1 (1.2–8.4) | 0.9 (0.5–1.6) | <0.001 |
| **Direct bilirubin (mg/dL)** | 2.3 (0.5–5.8) | 3.5 (0.8–6.2) | 0.4 (0.2–1.0) | 0.002 |
| **Indirect bilirubin (mg/dL)** | 0.6 (0.3–1.2) | 0.7 (0.3–1.2) | 0.2 (0.1–0.8) | 0.006 |
| **Exposure variables** | | | | |
| **Contact with hepatitis case** |  |  |  | 0.734 |
| No | 101 (81.5%) | 87 (80.6%) | 14 (87.5%) |  |
| Yes | 23 (18.5%) | 21 (19.4%) | 2 (12.5%) |  |
| **Access to sewage/treated water** |  |  |  | >0.999 |
| No | 10 (8.1%) | 9 (8.3%) | 1 (6.3%) |  |
| Yes | 114 (91.9%) | 99 (91.7%) | 15 (93.8%) |  |
| **Seafood** |  |  |  | 0.014 |
| No | 73 (58.9%) | 59 (54.6%) | 14 (87.5%) |  |
| Yes | 51 (41.1%) | 49 (45.4%) | 2 (12.5%) |  |
| **Tattoo or piercing** |  |  |  | 0.788 |
| No | 70 (56.5%) | 60 (55.6%) | 10 (62.5%) |  |
| Yes | 54 (43.5%) | 48 (44.4%) | 6 (37.5%) |  |
| **Acupuncture** |  |  |  | >0.999 |
| No | 120 (96.8%) | 104 (96.3%) | 16 (100.0%) |  |
| Yes | 4 (3.2%) | 4 (3.7%) | 0 (0.0%) |  |
| **Surgery** |  |  |  | 0.005 |
| No | 92 (74.2%) | 85 (78.7%) | 7 (43.8%) |  |
| Yes | 32 (25.8%) | 23 (21.3%) | 9 (56.3%) |  |
| **Dental procedure** |  |  |  | 0.587 |
| No | 73 (58.9%) | 65 (60.2%) | 8 (50.0%) |  |
| Yes | 51 (41.1%) | 43 (39.8%) | 8 (50.0%) |  |
| **Transplant** |  |  |  | 0.129 |
| No | 123 (99.2%) | 108 (100.0%) | 15 (93.8%) |  |
| Yes | 1 (0.8%) | 0 (0.0%) | 1 (6.3%) |  |
| **Hemodialysis** |  |  |  | >0.999 |
| No | 124 (100.0%) | 108 (100.0%) | 16 (100.0%) |  |
| Yes | 0 (0.0%) | 0 (0.0%) | 0 (0.0%) |  |
| **Biological accident** |  |  |  | >0.999 |
| No | 117 (94.4%) | 102 (94.4%) | 15 (93.8%) |  |
| Yes | 7 (5.6%) | 6 (5.6%) | 1 (6.3%) |  |
| **Behavioral** **variables** | | | | |
| **Sexual orientation** |  |  |  | 0.004 |
| Heterosexual | 72 (64.3%) | 56 (58.3%) | 16 (100.0%) |  |
| Homosexual | 32 (28.6%) | 32 (33.3%) | 0 (0.0%) |  |
| Bisexual | 8 (7.1%) | 8 (8.3%) | 0 (0.0%) |  |
| **Men who have sex with men (MSM)** |  |  |  | 0.003 |
| Non-MSM | 86 (69.4%) | 70 (64.8%) | 16 (100.0%) |  |
| MSM | 38 (30.6%) | 38 (35.2%) | 0 (0.0%) |  |
| **Injectable drug use** |  |  |  | >0.999 |
| No | 122 (99.2%) | 106 (99.1%) | 16 (100.0%) |  |
| Yes | 1 (0.8%) | 1 (0.9%) | 0 (0.0%) |  |
| **Inhalable drug use** |  |  |  | >0.999 |
| No | 98 (80.3%) | 85 (80.2%) | 13 (81.3%) |  |
| Yes | 24 (19.7%) | 21 (19.8%) | 3 (18.8%) |  |
| **Alcohol consumption** |  |  |  | 0.087 |
| Never | 29 (23.4%) | 20 (18.5%) | 9 (56.3%) |  |
| Rarely | 37 (29.8%) | 33 (30.6%) | 4 (25.0%) |  |
| 1x/week | 28 (22.6%) | 26 (24.1%) | 2 (12.5%) |  |
| 2x/week | 18 (14.5%) | 17 (15.7%) | 1 (6.3%) |  |
| 3x/week | 5 (4.0%) | 5 (4.6%) | 0 (0.0%) |  |
| ≥4x/week | 7 (5.6%) | 7 (6.5%) | 0 (0.0%) |  |
| **Coinfections** | | | | |
| **Toxoplasmosis Anti-IgG** |  |  |  | <0.001 |
| Non-reactive | 87 (70.2%) | 82 (75.9%) | 5 (31.3%) |  |
| Indeterminate | 1 (0.8%) | 1 (0.9%) | 0 (0.0%) |  |
| Reactive | 36 (29.0%) | 25 (23.1%) | 11 (68.8%) |  |
| ^1^Continuous variables: median (Q1–Q3); categorical variables: n (%). | | | | |
| ^2^Fisher’s exact test for categorical variables; Mann–Whitney U test for continuous variables. | | | | |
