# Supplementary material for: Hepatitis E in suspected acute hepatitis cases in Brazil: A comparison with hepatitis A
Source: PLoS Negl Trop Dis. 2026 Oct 26;20(10):e0014834. doi: 10.1371/journal.pntd.0014834 (PMC13766607; doi:10.1371/journal.pntd.0014834)
Supplement: Supplementary file 2 [file pntd.0014834.s002.docx]

S2 Table . Demographic, clinical, and behavioral characteristics associated with anti-HEV IgG reactivity

| **Characteristic** | **Overall, N = 1720^1^** | **Anti-HEV IgG (−), N = 1631^1^** | **Anti-HEV IgG (+), N = 89^1^** | **p-value^2^** |
| --- | --- | --- | --- | --- |
| **Demographic data** | | | | |
| **Age group (years)** |  |  |  | <0.001 |
| 18-24 | 181 (10.5%) | 178 (10.9%) | 3 (3.4%) |  |
| 25-29 | 207 (12.0%) | 205 (12.6%) | 2 (2.2%) |  |
| 30-34 | 210 (12.2%) | 207 (12.7%) | 3 (3.4%) |  |
| 35-39 | 209 (12.2%) | 200 (12.3%) | 9 (10.1%) |  |
| 40-44 | 212 (12.3%) | 193 (11.8%) | 19 (21.3%) |  |
| 45-49 | 163 (9.5%) | 154 (9.4%) | 9 (10.1%) |  |
| 50-54 | 152 (8.8%) | 143 (8.8%) | 9 (10.1%) |  |
| 55-59 | 119 (6.9%) | 111 (6.8%) | 8 (9.0%) |  |
| 60-64 | 106 (6.2%) | 100 (6.1%) | 6 (6.7%) |  |
| ≥65 | 161 (9.4%) | 140 (8.6%) | 21 (23.6%) |  |
| **Age (years)** | 41.0 (30.5–53.0) | 40.0 (30.0–52.0) | 49.0 (40.0–63.0) | <0.001 |
| **Sex at birth** |  |  |  | >0.999 |
| Female | 729 (42.4%) | 691 (42.4%) | 38 (42.7%) |  |
| Male | 991 (57.6%) | 940 (57.6%) | 51 (57.3%) |  |
| **Gender identity** |  |  |  | 0.609 |
| Cis man | 944 (56.8%) | 896 (56.8%) | 48 (55.8%) |  |
| Cis woman | 705 (42.4%) | 668 (42.4%) | 37 (43.0%) |  |
| Trans man | 1 (0.1%) | 1 (0.1%) | 0 (0.0%) |  |
| Trans woman | 9 (0.5%) | 8 (0.5%) | 1 (1.2%) |  |
| Nonbinary | 4 (0.2%) | 4 (0.3%) | 0 (0.0%) |  |
| **Race** |  |  |  | 0.208 |
| White | 706 (58.8%) | 657 (58.1%) | 49 (71.0%) |  |
| Black | 127 (10.6%) | 123 (10.9%) | 4 (5.8%) |  |
| Mixed | 352 (29.3%) | 336 (29.7%) | 16 (23.2%) |  |
| Asian | 15 (1.3%) | 15 (1.3%) | 0 (0.0%) |  |
| **Geographical characteristics of the place of residence** |  |  |  | 0.525 |
| Urban area | 1,594 (92.8%) | 1,513 (92.9%) | 81 (91.0%) |  |
| Rural area | 124 (7.2%) | 116 (7.1%) | 8 (9.0%) |  |
| **Region of residence** |  |  |  | 0.007 |
| North | 244 (14.2%) | 239 (14.7%) | 5 (5.6%) |  |
| Northeast | 212 (12.3%) | 207 (12.7%) | 5 (5.6%) |  |
| Central-West | 125 (7.3%) | 118 (7.2%) | 7 (7.9%) |  |
| Southeast | 754 (43.8%) | 711 (43.6%) | 43 (48.3%) |  |
| South | 385 (22.4%) | 356 (21.8%) | 29 (32.6%) |  |
| **Marital status** |  |  |  | 0.121 |
| Single | 724 (42.9%) | 694 (43.4%) | 30 (33.7%) |  |
| Married | 626 (37.1%) | 587 (36.7%) | 39 (43.8%) |  |
| Stable union | 157 (9.3%) | 152 (9.5%) | 5 (5.6%) |  |
| Separated | 27 (1.6%) | 25 (1.6%) | 2 (2.2%) |  |
| Divorced | 86 (5.1%) | 79 (4.9%) | 7 (7.9%) |  |
| Widowed | 69 (4.1%) | 63 (3.9%) | 6 (6.7%) |  |
| **Inclusion criteria** | | | | |
| **Inclusion criteria** |  |  |  | 0.027 |
| Anicteric asymptomatic | 240 (14.0%) | 232 (14.2%) | 8 (9.0%) |  |
| Anicteric symptomatic | 641 (37.3%) | 596 (36.5%) | 45 (50.6%) |  |
| Icteric symptomatic | 839 (48.8%) | 803 (49.2%) | 36 (40.4%) |  |
| **Biochemical laboratory data** | | | | |
| **ALT (U/L)** | 149.0 (54.5–400.0) | 150.0 (55.0–399.0) | 119.0 (52.0–413.0) | 0.595 |
| **AST (U/L)** | 100.0 (44.0–280.0) | 101.5 (43.5–284.5) | 89.0 (50.0–213.0) | 0.597 |
| **GGT (U/L)** | 200.5 (82.0–446.0) | 198.0 (81.0–438.0) | 249.5 (115.5–534.5) | 0.060 |
| **Alkaline phosphatase (U/L)** | 138.0 (93.0–234.0) | 137.0 (92.0–228.0) | 157.0 (98.5–299.5) | 0.093 |
| **Total bilirubin (mg/dL)** | 0.8 (0.4–4.4) | 0.8 (0.4–4.5) | 0.9 (0.4–2.8) | 0.691 |
| **Direct bilirubin (mg/dL)** | 0.4 (0.2–3.7) | 0.4 (0.2–3.8) | 0.6 (0.2–2.1) | 0.991 |
| **Indirect bilirubin (mg/dL)** | 0.3 (0.2–0.7) | 0.3 (0.2–0.7) | 0.3 (0.1–0.6) | 0.318 |
| **Exposure variables** | | | | |
| **Contact with hepatitis case** |  |  |  | 0.477 |
| No | 1,625 (94.6%) | 1,542 (94.7%) | 83 (93.3%) |  |
| Yes | 93 (5.4%) | 87 (5.3%) | 6 (6.7%) |  |
| **Access to sewage/treated water** |  |  |  | 0.652 |
| No | 264 (15.3%) | 249 (15.3%) | 15 (16.9%) |  |
| Yes | 1,456 (84.7%) | 1,382 (84.7%) | 74 (83.1%) |  |
| **Seafood** |  |  |  | 0.002 |
| No | 1,351 (78.6%) | 1,270 (78.0%) | 81 (91.0%) |  |
| Yes | 367 (21.4%) | 359 (22.0%) | 8 (9.0%) |  |
| **Tattoo or piercing** |  |  |  | 0.480 |
| No | 1,186 (69.1%) | 1,121 (68.9%) | 65 (73.0%) |  |
| Yes | 531 (30.9%) | 507 (31.1%) | 24 (27.0%) |  |
| Missing | 3 | 3 | 0 |  |
| **Acupuncture** |  |  |  | 0.400 |
| No | 1,596 (93.0%) | 1,515 (93.1%) | 81 (91.0%) |  |
| Yes | 121 (7.0%) | 113 (6.9%) | 8 (9.0%) |  |
| **Surgery** |  |  |  | 0.089 |
| No | 1,095 (63.7%) | 1,046 (64.2%) | 49 (55.1%) |  |
| Yes | 623 (36.3%) | 583 (35.8%) | 40 (44.9%) |  |
| **Dental procedure** |  |  |  | 0.745 |
| No | 877 (51.0%) | 830 (51.0%) | 47 (52.8%) |  |
| Yes | 841 (49.0%) | 799 (49.0%) | 42 (47.2%) |  |
| **Transplant** |  |  |  | 0.188 |
| No | 1,666 (97.0%) | 1,582 (97.1%) | 84 (94.4%) |  |
| Yes | 52 (3.0%) | 47 (2.9%) | 5 (5.6%) |  |
| **Hemodialysis** |  |  |  | 0.417 |
| No | 1,684 (98.0%) | 1,598 (98.1%) | 86 (96.6%) |  |
| Yes | 34 (2.0%) | 31 (1.9%) | 3 (3.4%) |  |
| **Biological accident** |  |  |  | 0.727 |
| No | 1,673 (97.4%) | 1,585 (97.3%) | 88 (98.9%) |  |
| Yes | 45 (2.6%) | 44 (2.7%) | 1 (1.1%) |  |
| **Behavioral variables** | | | | |
| **Sexual orientation** |  |  |  | 0.008 |
| Heterosexual | 1,396 (85.3%) | 1,316 (84.7%) | 80 (96.4%) |  |
| Homosexual | 215 (13.1%) | 212 (13.6%) | 3 (3.6%) |  |
| Bisexual | 26 (1.6%) | 26 (1.7%) | 0 (0.0%) |  |
| **Men who have sex with men (MSM)** |  |  |  | 0.005 |
| Non-MSM | 1,502 (87.3%) | 1,416 (86.8%) | 86 (96.6%) |  |
| MSM | 218 (12.7%) | 215 (13.2%) | 3 (3.4%) |  |
| **Injectable drug use** |  |  |  | >0.999 |
| No | 1,663 (97.5%) | 1,576 (97.5%) | 87 (97.8%) |  |
| Yes | 42 (2.5%) | 40 (2.5%) | 2 (2.2%) |  |
| **Inhalable drug use** |  |  |  | 0.280 |
| No | 1,450 (85.3%) | 1,370 (85.0%) | 80 (89.9%) |  |
| Yes | 250 (14.7%) | 241 (15.0%) | 9 (10.1%) |  |
| **Alcohol consumption** |  |  |  | 0.120 |
| Never | 668 (38.9%) | 632 (38.8%) | 36 (40.4%) |  |
| Rarely | 499 (29.1%) | 477 (29.3%) | 22 (24.7%) |  |
| 1x/week | 214 (12.5%) | 205 (12.6%) | 9 (10.1%) |  |
| 2x/week | 128 (7.5%) | 123 (7.6%) | 5 (5.6%) |  |
| 3x/week | 65 (3.8%) | 63 (3.9%) | 2 (2.2%) |  |
| ≥4x/week | 142 (8.3%) | 127 (7.8%) | 15 (16.9%) |  |
| **Coinfections** | | | | |
| **Acute Hepatitis A (AHA)** |  |  |  | 0.040 |
| Absent | 1,612 (93.7%) | 1,524 (93.4%) | 88 (98.9%) |  |
| Present | 108 (6.3%) | 107 (6.6%) | 1 (1.1%) |  |
| **Anti-HAV IgG** |  |  |  | <0.001 |
| Non-reactive | 368 (21.4%) | 365 (22.4%) | 3 (3.4%) |  |
| Reactive | 1,349 (78.6%) | 1,263 (77.6%) | 86 (96.6%) |  |
| **Anti-HBc total** |  |  |  | 0.012 |
| Non-reactive | 1,395 (81.3%) | 1,332 (81.9%) | 63 (70.8%) |  |
| Reactive | 321 (18.7%) | 295 (18.1%) | 26 (29.2%) |  |
| **IgG Anti-Toxoplasmosis** |  |  |  | <0.001 |
| Non-reactive | 787 (46.0%) | 763 (47.0%) | 24 (27.3%) |  |
| Reactive | 924 (54.0%) | 860 (53.0%) | 64 (72.7%) |  |
| **Leptospirosis** |  |  |  | 0.002 |
| Non-reactive | 1,701 (98.9%) | 1,617 (99.1%) | 84 (94.4%) |  |
| Reactive | 19 (1.1%) | 14 (0.9%) | 5 (5.6%) |  |
| ^1^Continuous variables: median (Q1–Q3); categorical variables: n (%). | | | | |
| ^2^Fisher’s exact test for categorical variables; Mann–Whitney U test for continuous variables. | | | | |
