# Supplementary material for: Hepatitis E in suspected acute hepatitis cases in Brazil: A comparison with hepatitis A
Source: PLoS Negl Trop Dis. 2026 Oct 26;20(10):e0014834. doi: 10.1371/journal.pntd.0014834 (PMC13766607; doi:10.1371/journal.pntd.0014834)
Supplement: Supplementary file 3 [file pntd.0014834.s003.docx]

**Subject: Response letter addressing technical check requests and reviewer comments for Manuscript ID PNTD-D-26-00195R1**

**Dear PLOS NTD team,**

We are pleased to submit this response letter in connection with our revised manuscript, **"Clinical and Epidemiological Comparison of Hepatitis A and E Infections in Brazil"** (Manuscript ID: **PNTD-D-26-00195R1**). The first two files submitted with this response, namely the present **Response Letter** and the **Response to Reviewers**, jointly address all questions, requests, and comments raised by the PLOS Neglected Tropical Diseases editorial team and by the reviewers during the technical check and peer-review process.

Kind regards,

**João Renato Rebello Pinho, MD, PhD**
On behalf of all authors

__________________________________________________________________________

**Response to the email received on Tuesday, August 18, 2026, at 03:46**

**Subject:** Re: Manuscript ID PNTD-D-26-00195R1

**Dear Ms. Venkatesan**,

Thank you for your email and for the careful technical review of our submission, **"Clinical and Epidemiological Comparison of Hepatitis A and E Infections in Brazil"** (Manuscript ID: **PNTD-D-26-00195R1**). We appreciate the opportunity to submit this response letter and to clarify the actions taken in relation to all communications received concerning this manuscript submission.

Below, we provide point-by-point responses to each item raised and indicate how the manuscript files, supporting materials, and submission record have been revised accordingly.

1. Removal of duplicate and unnecessary files from the submission inventory.

*The submission inventory has been carefully reviewed, and all duplicate, outdated, or unnecessary files have been removed. Only the files relevant to the current revised version of the manuscript have been retained in Editorial Manager. These files are:*

1. *Manuscript*
2. *Figure 1*
3. *Figure 2*
4. *Supporting Information*
5. *Map Copyright*
6. *Manuscript Source*
7. *Response to Reviewers*
8. *Response letter (present file)*
9. *Revised Manuscript with Track Changes*
2. Correction of the figure inventory and manuscript citations, ensuring consistency between figure files, legends, and in-text citations.

*The list of files currently retained in the submission inventory is shown above. We have reviewed the figure inventory and confirmed that all figure files retained in the submission have corresponding in-text citations and legends in the revised manuscript. The file submitted previously that was interpreted in the submission inventory as Figure 2 but did not correspond to a figure cited in the manuscript, has been removed from the inventory. The current Figure 2 file retained in the submission corresponds to the figure cited in the revised manuscript and is accompanied by the appropriate legend. The information requested in item 4 below regarding the source of the map base layer and its licensing terms has also been provided.*

3. Deposition of the study dataset in an appropriate public repository and updating the Data Availability Statement accordingly.

*This issue has been addressed. The minimal dataset necessary to replicate the study findings has been deposited in the Open Science Framework (OSF) repository. The manuscript has been updated accordingly, and the Data Availability Statement now appears on page 14, at the end of the Statistical analysis subsection, before the Results section, and states: “The dataset supporting the findings of this study has been deposited in the Open Science Framework (OSF) repository (https://osf.io/7gjsc).”*

4. Provision of the requested information regarding Figure 2, including the source of the map shapefile/base layer and its licensing terms, ensuring compliance with PLOS copyright and CC BY 4.0 requirements.

*This issue has been addressed. The requested information regarding the source of the map base layer and its licensing terms corresponds to file 7, “Map Copyright,” described in item 1 of the present response. This file includes the direct link to the source used for the boundaries of Brazil and its different geographic regions, as well as the corresponding terms of use/license information for the base layer. It has been retained in the submission inventory to document the source and copyright/license information for Figure 2.*

The revised files have been uploaded in Editorial Manager, and the submission has been re-approved.

Thank you again for your assistance. We appreciate your support and look forward to the next stage of the review process.

Kind regards,

**João Renato Rebello Pinho, MD, PhD**
On behalf of all authors

-----Mensagem original-----
De: em.pntd.0.9d840b.7c6696f5@editorialmanager.com <em.pntd.0.9d840b.7c6696f5@editorialmanager.com> Em nome de PLOS Neglected Tropical Diseases
Enviada em: **terça-feira, 18 de agosto de 2026 03:46**
Para: João Renato Rebello Pinho <joao.pinho@einstein.br>
Assunto: PLOS Neglected Tropical Diseases Technical Check requests for your submission PNTD-D-26-00195R1 - [EMID:b19200d68711a4cb]

--------------------------------------------------------------------------------------------------------

Manuscript ID: PNTD-D-26-00195R1

Dear Dr Pinho,

Thank you for submitting your paper, "Clinical and Epidemiological Comparison of Hepatitis A and E Infections in Brazil", to PLOS Neglected Tropical Diseases. We have checked over your submission and, before we can assign your manuscript to the Editor, we ask that you first please make the following modifications:

Please action the point below so that it can proceed with the review process.

1) We note that your Manuscript files are duplicated on your submission. Please remove any unnecessary or old files from your revision, and make sure that only those relevant to the current version of the manuscript are included.

2) Please ensure that all Figure files have corresponding citations and legends within the manuscript. Currently, Figure 2 in your submission file inventory does not have an in-text citation. If the figure is no longer to be included as part of the submission, please remove it from the file inventory.

3) Thank you for stating "The data that support the findings of this study are publicly available from: <https://nam10.safelinks.protection.outlook.com/?url=https%3A%2F%2Fdocs.google.com%2Fspreadsheets%2Fd%2F1qQuGfPyWmP5AkW2oNDqbdI9TBbHTAyRR%2Fedit%3Fusp%3Ddrive_link%26ouid%3D116910710240750571110%26rtpof%3Dtrue%26sd%3Dtrue&data=05%7C02%7Cjoao.pinho%40einstein.br%7C766efaeb132f4ca35cd408defcf44f85%7C6dc28345304849c1becad5cad28a8f77%7C0%7C0%7C639226323424888525%7CUnknown%7CTWFpbGZsb3d8eyJFbXB0eU1hcGkiOnRydWUsIlYiOiIwLjAuMDAwMCIsIlAiOiJXaW4zMiIsIkFOIjoiTWFpbCIsIldUIjoyfQ%3D%3D%7C0%7C%7C%7C&sdata=nTnoyrsJY6cyyc0iE%2B2F93KZkTyPUvFBu30ufVz7U4o%3D&reserved=0>

Unfortunately, Google Drive does not qualify as an acceptable data repository according to PLOS's standards.

At this time, please upload the minimal data set necessary to replicate your study's findings to a stable, public repository (such as figshare or Dryad) and provide us with the relevant URLs, DOIs, or accession numbers that may be used to access these data. For a list of recommended repositories and additional information on PLOS standards for data deposition, please see <https://nam10.safelinks.protection.outlook.com/?url=https%3A%2F%2Fjournals.plos.org%2Fplosone%2Fs%2Frecommended-repositories&data=05%7C02%7Cjoao.pinho%40einstein.br%7C766efaeb132f4ca35cd408defcf44f85%7C6dc28345304849c1becad5cad28a8f77%7C0%7C0%7C639226323424913722%7CUnknown%7CTWFpbGZsb3d8eyJFbXB0eU1hcGkiOnRydWUsIlYiOiIwLjAuMDAwMCIsIlAiOiJXaW4zMiIsIkFOIjoiTWFpbCIsIldUIjoyfQ%3D%3D%7C0%7C%7C%7C&sdata=8qCrT%2BplLnFNs%2FXn%2FbxFa%2FWzxfiRM37svsA1GkxpZ%2FE%3D&reserved=0>

4) Some material included in your submission may be copyrighted. According to PLOS’s copyright policy, authors who use figures or other material (e.g., graphics, clipart, maps) from another author or copyright holder must demonstrate or obtain permission to publish this material under the Creative Commons Attribution 4.0 International (CC BY 4.0) License used by PLOS journals. Please closely review the details of PLOS’s copyright requirements here: PLOS Licenses and Copyright. If you need to request permissions from a copyright holder, you may use PLOS's Copyright Content Permission form.

Please respond directly to this email and provide any known details concerning your material's license terms and permissions required for reuse, even if you have not yet obtained copyright permissions or are unsure of your material's copyright compatibility. Once you have responded and addressed all other outstanding technical requirements, you may resubmit your manuscript within Editorial Manager.

Potential Copyright Issues:

[Figure 2]: please (a) provide a direct link to the base layer of the map (i.e., the country or region border shape) and ensure this is also included in the figure legend; and (b) provide a link to the terms of use / license information for the base layer image or shapefile. We cannot publish proprietary or copyrighted maps (e.g. Google Maps, Mapquest) and the terms of use for your map base layer must be compatible with our CC BY 4.0 license.

Note: if you created the map in a software program like R or ArcGIS, please locate and indicate the source of the basemap shapefile onto which data has been plotted.

If your map was obtained from a copyrighted source please amend the figure so that the base map used is from an openly available source. Alternatively, please provide explicit written permission from the copyright holder granting you the right to publish the material under our CC BY 4.0 license.

If you are unsure whether you can use a map or not, please do reach out and we will be able to help you. The following websites are good examples of where you can source open access or public domain maps:

* U.S. Geological Survey (USGS) - All maps are in the public domain. (<https://nam10.safelinks.protection.outlook.com/?url=http%3A%2F%2Fwww.usgs.gov%2F&data=05%7C02%7Cjoao.pinho%40einstein.br%7C766efaeb132f4ca35cd408defcf44f85%7C6dc28345304849c1becad5cad28a8f77%7C0%7C0%7C639226323424928126%7CUnknown%7CTWFpbGZsb3d8eyJFbXB0eU1hcGkiOnRydWUsIlYiOiIwLjAuMDAwMCIsIlAiOiJXaW4zMiIsIkFOIjoiTWFpbCIsIldUIjoyfQ%3D%3D%7C0%7C%7C%7C&sdata=zc6TVWDEfj%2BPjAHtG2PfGyR0mpxvvtwBhrflna99Cjo%3D&reserved=0>)

* PlaniGlobe - All maps are published under a Creative Commons license so please cite “PlaniGlobe, <https://nam10.safelinks.protection.outlook.com/?url=http%3A%2F%2Fwww.planiglobe.com%2F&data=05%7C02%7Cjoao.pinho%40einstein.br%7C766efaeb132f4ca35cd408defcf44f85%7C6dc28345304849c1becad5cad28a8f77%7C0%7C0%7C639226323424941204%7CUnknown%7CTWFpbGZsb3d8eyJFbXB0eU1hcGkiOnRydWUsIlYiOiIwLjAuMDAwMCIsIlAiOiJXaW4zMiIsIkFOIjoiTWFpbCIsIldUIjoyfQ%3D%3D%7C0%7C%7C%7C&sdata=LWEp16MH9o3R%2BP97dbqKQfMOT8PAMgplV7oxqOT0eds%3D&reserved=0>, CC BY 2.0” in the image credit after the caption. (<https://nam10.safelinks.protection.outlook.com/?url=http%3A%2F%2Fwww.planiglobe.com%2F%3Flang%3Denl&data=05%7C02%7Cjoao.pinho%40einstein.br%7C766efaeb132f4ca35cd408defcf44f85%7C6dc28345304849c1becad5cad28a8f77%7C0%7C0%7C639226323424953550%7CUnknown%7CTWFpbGZsb3d8eyJFbXB0eU1hcGkiOnRydWUsIlYiOiIwLjAuMDAwMCIsIlAiOiJXaW4zMiIsIkFOIjoiTWFpbCIsIldUIjoyfQ%3D%3D%7C0%7C%7C%7C&sdata=B7GnDhEV2TLLa%2B1NE9ImoK9UqRixuRdShwccExQgvoA%3D&reserved=0>)

* Natural Earth - All maps are public domain. (<https://nam10.safelinks.protection.outlook.com/?url=http%3A%2F%2Fwww.naturalearthdata.com%2Fabout%2Fterms-of-use%2F&data=05%7C02%7Cjoao.pinho%40einstein.br%7C766efaeb132f4ca35cd408defcf44f85%7C6dc28345304849c1becad5cad28a8f77%7C0%7C0%7C639226323424965850%7CUnknown%7CTWFpbGZsb3d8eyJFbXB0eU1hcGkiOnRydWUsIlYiOiIwLjAuMDAwMCIsIlAiOiJXaW4zMiIsIkFOIjoiTWFpbCIsIldUIjoyfQ%3D%3D%7C0%7C%7C%7C&sdata=aGPJ%2BQkjSE%2Fc1zy5rfxBuvPHoTwrXWIJHbeyWI%2B6IAc%3D&reserved=0>)"

Once you have made the necessary changes in Editorial Manager, please re-approve the submission by following the same steps as before.

Please resolve these issues and resubmit your manuscript within 30 days. If you need more time, please email the journal office at [plosntds@plos.org](mailto:plosntds@plos.org). We are happy to grant extensions of up to one month past this due date. If we do not hear from you within 30 days, we will withdraw your manuscript on your behalf.

If you have any questions or require assistance, please don't hesitate to get in touch. To help us minimize inbox volumes, we kindly ask that you not reply to this email simply to confirm you have resubmitted your revisions.

Kind regards,

Nithya Venkatesan

PLOS Neglected Tropical Diseases

[plosntds@plos.org](mailto:plosntds@plos.org)

Author guidelines: <https://nam10.safelinks.protection.outlook.com/?url=https%3A%2F%2Fjournals.plos.org%2Fplosntds%2Fs%2Fsubmission-guidelines&data=05%7C02%7Cjoao.pinho%40einstein.br%7C766efaeb132f4ca35cd408defcf44f85%7C6dc28345304849c1becad5cad28a8f77%7C0%7C0%7C639226323424977705%7CUnknown%7CTWFpbGZsb3d8eyJFbXB0eU1hcGkiOnRydWUsIlYiOiIwLjAuMDAwMCIsIlAiOiJXaW4zMiIsIkFOIjoiTWFpbCIsIldUIjoyfQ%3D%3D%7C0%7C%7C%7C&sdata=GDWv%2BrnwOXpAE9uRO2iebQbxU9VyEfBoZFAFWHV9fcA%3D&reserved=0>

____________________________________________________________________________

**Response to the email received on Friday, August 7, 2026, at 02:00**

**De:** em.pntd.0.9d45c3.7de8dc0f@editorialmanager.com <em.pntd.0.9d45c3.7de8dc0f@editorialmanager.com> em nome de PLOS Neglected Tropical Diseases <em@editorialmanager.com>
**Enviado:** **sexta-feira, 7 de agosto de 2026 02:00**
**Para:** João Renato Rebello Pinho <joao.pinho@einstein.br>
**Assunto:** PLOS Neglected Tropical Diseases Technical Check requests for your submission PNTD-D-26-00195R1 - [EMID:8e80519bf0c3509d]

**Dear Ms. Samidurai,**

Thank you for your email and for the technical check of our revised submission, **"Clinical and Epidemiological Comparison of Hepatitis A and E Infections in Brazil"** (Manuscript ID: **PNTD-D-26-00195R1**). We appreciate the opportunity to clarify the actions taken in response to the points raised in your message.

Below, we provide point-by-point responses and indicate where each requested item has been addressed in the current submission and in the response provided above.

-------------------------------------------------------------------------------------------------------

Manuscript ID: PNTD-D-26-00195R1

Dear Dr Pinho,

Thank you for submitting your paper, "Clinical and Epidemiological Comparison of Hepatitis A and E Infections in Brazil", to PLOS Neglected Tropical Diseases. We have checked over your submission and, before we can assign your manuscript to the Editor, we ask that you first please make the following modifications:

1) We ask that a manuscript source file is provided at Revision. Please upload your manuscript file as a .doc, .docx, .rtf or .tex. If you are providing a .tex file, please upload it under the item type ‘LaTeX Source File’ and leave your .pdf version as the item type ‘Manuscript’.

*This request has been addressed as described in the response provided above. The manuscript source file has been included among the retained submission files, specifically as “Manuscript Source”.*

2) We have noticed that you have uploaded Supporting Information files, but you have not included a list of legends. Please add a full list of legends for your Supporting Information files after the references list.

*This request has been addressed. A full list of legends for the Supporting Information files has been added after the references, at the beginning of page 43 of the revised manuscript.*

3) We notice that your supplementary Tables are included in the manuscript file. Please remove them and upload them with the file type 'Supporting Information'. Please ensure that each Supporting Information file has a legend listed in the manuscript after the references list.

*This request has been addressed. The supplementary tables have been removed from the manuscript file and uploaded separately with the file type “Supporting Information.” Their corresponding legends have been included in the manuscript after the references, as described in the response to item 2 above.*

4) Please amend your detailed Financial Disclosure statement. This is published with the article. It must therefore be completed in full sentences and contain the exact wording you wish to be published.
1) State the initials, alongside each funding source, of each author to receive each grant. For example: "This work was supported by the National Institutes of Health (####### to AM; ###### to CJ) and the National Science Foundation (###### to AM)."
2) State what role the funders took in the study. If the funders had no role in your study, please state: "The funders had no role in study design, data collection and analysis, decision to publish, or preparation of the manuscript.".
If you did not receive any funding for this study, please simply state: “The authors received no specific funding for this work.”
*Thank you for your comment. We have revised the Financial Disclosure statement to specify the funding source and the author associated with each grant. The updated statement now fully complies with the journal’s requirements and reflects the exact wording intended for publication. A text was added on page 43:*

*“****Funding***

*This study was supported by Hospital Israelita Albert Einstein through the Program for Supporting the Institutional Development of the Public Health System (PROADI‐SUS), supported by the Ministry of Health of Brazil. Some authors are employees of Hospital Israelita Albert Einstein and the Brazilian Ministry of Health. The study was designed conjointly accordingly to research priorities chosen by the Ministry of Health of Brazil and the authors are responsible for data collection and analysis, decision to publish, and preparation of the manuscript.*

*KGO is a graduate student in the Graduate Program in Gastroenterology Sciences at Faculdade de Medicine, Universidade de São Paulo.*

*JRRP receives a fellowship from Conselho Nacional de Desenvolvimento Científico e Tecnológico (CNPq) - Bolsista de produtividade).”*

Once you have made the necessary changes in Editorial Manager, please re-approve the submission by following the same steps as before.

Please resolve these issues and resubmit your manuscript within 30 days. If you need more time, please email the journal office at plosntds@plos.org. We are happy to grant extensions of up to one month past this due date. If we do not hear from you within 30 days, we will withdraw your manuscript on your behalf.

If you have any questions or require assistance, please don't hesitate to get in touch. To help us minimize inbox volumes, we kindly ask that you not reply to this email simply to confirm you have resubmitted your revisions.

Kind regards,

Anitha Samidurai
PLOS Neglected Tropical Diseases
plosntds@plos.org

All requested changes described in your technical check have therefore been completed in the revised submission. The manuscript files, Supporting Information files, legends, and Financial Disclosure statement have been updated accordingly in Editorial Manager.

Thank you again for your assistance. We appreciate your guidance and hope that the revised submission now meets all technical requirements for further processing.

Kind regards,

**João Renato Rebello Pinho, MD, PhD**
On behalf of all authors

**Response to the email received on Friday, August 7, 2026, at 01:58**

**Dear Ms. Samidurai,**

Thank you for your email regarding the potential copyright issue related to Figure 2 in our submission, **"Clinical and Epidemiological Comparison of Hepatitis A and E Infections in Brazil"** (Manuscript ID: **PNTD-D-26-00195R1**). This point has been addressed in the responses provided above and in the revised submission files.

Specifically, the requested information regarding the source of the map base layer and its licensing terms has been provided in file 4, **“Map Copyright,”** listed among the retained submission files. This file includes the direct link to the source used for the boundaries of Brazil and its different geographic regions, as well as the corresponding terms of use/license information for the base layer.

The current Figure 2 file retained in the submission corresponds to the figure cited in the revised manuscript and is accompanied by the appropriate legend. The previously submitted file that had been interpreted in the submission inventory as Figure 2, but did not correspond to a figure cited in the manuscript, has been removed from the inventory.

We hope that the information now provided resolves the potential copyright issue raised for Figure 2 and confirms that the map base layer is appropriately documented for compatibility with PLOS requirements.

Kind regards,

**João Renato Rebello Pinho, MD, PhD**
On behalf of all authors

**De:** em.pntd.19293b.9d45c0.8b04d553@editorialmanager.com <em.pntd.19293b.9d45c0.8b04d553@editorialmanager.com> em nome de Anitha Samidurai <em@editorialmanager.com>
**Enviado:** **sexta-feira, 7 de agosto de 2026 01:58**
**Para:** João Renato Rebello Pinho <joao.pinho@einstein.br>
**Assunto:** Response required: PLOS Neglected Tropical Diseases Potential copyright issues for your submission PNTD-D-26-00195R1 - [EMID:2b3808d2da3868d9]

--------------------------------------------------------------------------------------------------------

Re: PNTD-D-26-00195R1
Clinical and Epidemiological Comparison of Hepatitis A and E Infections in Brazil

Dear Dr Pinho,
Thank you for your submission to PLOS Neglected Tropical Diseases entitled, "Clinical and Epidemiological Comparison of Hepatitis A and E Infections in Brazil".

Your manuscript is currently being checked and you will receive a separate email should you be required to make any immediate changes. Due to some of the content in your manuscript, however, we would like to go ahead and raise some potential copyright issues.

Please respond directly to this email and provide any known details concerning your material's license terms and permissions required for reuse, even if you have not yet obtained copyright permissions or are unsure of your material's copyright compatibility. **Note that we will not be able to process your submission further until we receive a response.**

**Copyright Policy**

Authors who use figures or other material (e.g., graphics, clipart, maps) from another author or copyright holder must obtain permission to publish this material under the [Creative Commons Attribution 4.0 International](https://nam10.safelinks.protection.outlook.com/?url=https%3A%2F%2Ftrack.editorialmanager.com%2FCL0%2Fhttps%3A%252F%252Fcreativecommons.org%252Flicenses%252Fby%252F4.0%252F%2F1%2F010f019fda95fac6-94271fd7-397d-4486-996e-ae9d246333ed-000000%2FEYwzla9iRHp-8xxfZ4x6i4TOvMFuSEbCKWAu3t3oKiE%3D258&data=05%7C02%7Cjoao.pinho%40einstein.br%7C3240cf0785f34685fc7908def4407e60%7C6dc28345304849c1becad5cad28a8f77%7C0%7C0%7C639216755629680591%7CUnknown%7CTWFpbGZsb3d8eyJFbXB0eU1hcGkiOnRydWUsIlYiOiIwLjAuMDAwMCIsIlAiOiJXaW4zMiIsIkFOIjoiTWFpbCIsIldUIjoyfQ%3D%3D%7C0%7C%7C%7C&sdata=7BB4Pxv%2F16J5IrIP5o0uew6uaZgW1pX6GXavg5ZsGC8%3D&reserved=0) (CC BY 4.0) License used by PLOS journals.

Under this license, authors retain ownership of the copyright for their article but allow anyone to download, reuse, reprint, modify, distribute, and/or copy articles and their content for any reason, even commercially, so long as the original authors and source are cited. Permission must therefore explicitly state that the copyright holder or representative will allow publication of the material under a CC BY 4.0 license.

Please note that purchasing copyright use is unlikely to meet this requirement, as many journals and publishers restrict the terms of purchased copyright use in ways that do not accommodate open access publication.

If you need to obtain permission to use a figure or other material in your manuscript, please fill out the first page of the [PLOS Content Copyright Permission Form](https://nam10.safelinks.protection.outlook.com/?url=https%3A%2F%2Ftrack.editorialmanager.com%2FCL0%2Fhttps%3A%252F%252Fstorage.googleapis.com%252Fplos-published-prod%252Fc4ff%252FContent%252520Copyright%252520Permission%252520Form.pdf%253FX-Goog-Algorithm%3DGOOG4-RSA-SHA256%2526X-Goog-Credential%3Dwombat-sa%252540plos-prod.iam.gserviceaccount.com%25252F20240108%25252Fauto%25252Fstorage%25252Fgoog4_request%2526X-Goog-Date%3D20240108T150748Z%2526X-Goog-Expires%3D86400%2526X-Goog-SignedHeaders%3Dhost%2526X-Goog-Signature%3Db07b95e5588eeb11255a4bea46b62f15e519fdce2c73f7863c7f5d8284b4d3bbcced61f4fa81de2dbee25c64a14124ff5c9c5432a4453c00c8528886f5b54c0d6583009c6741de3cda068b08bf1335cd17c9c2abf6d29bb532072f505fe2f45b1ac7dcfa7392a0772f50273283d6e3fc9daa29b4edf0816314d203507b32fd61effcd64a7daba9282e4cca6c2003bc8687f7b34f6a6a2e2ea3c03e417a142845faa95be0607e9bf1690855080d59a4edeeea496b532723387f2d97a47ae64140fbcaf22f6d344651c279130df68627d9d8255e2c9fa8a509353d1d6bd232dea355e1091f847fbb6f708b9fbae6a16788325d114ffe178b010b2f990a88622a5b%2F1%2F010f019fda95fac6-94271fd7-397d-4486-996e-ae9d246333ed-000000%2FMiDJreaM38LZI-tQkhwqhcZbYAcgkbQsg1vBQO18FjA%3D258&data=05%7C02%7Cjoao.pinho%40einstein.br%7C3240cf0785f34685fc7908def4407e60%7C6dc28345304849c1becad5cad28a8f77%7C0%7C0%7C639216755629703589%7CUnknown%7CTWFpbGZsb3d8eyJFbXB0eU1hcGkiOnRydWUsIlYiOiIwLjAuMDAwMCIsIlAiOiJXaW4zMiIsIkFOIjoiTWFpbCIsIldUIjoyfQ%3D%3D%7C0%7C%7C%7C&sdata=YA8kHgFhGZg%2Bu52s%2BT%2BiEP%2BZdOBkUzI%2BQu21qiNqTV0%3D&reserved=0) and ask the relevant copyright holder, office, or representative to complete the second page. Return the complete, signed copy to the journal via email. Permission is not required from PLOS or authors to reuse any content previously published in a PLOS journal as long as proper attribution is provided.

--------------------------------------------------

**Potential Copyright Issues:**

1) Figure 2. Please (a) provide a direct link to the base layer of the map (i.e., the country or region border shape) and ensure this is also included in the figure legend; and (b) provide a link to the terms of use / license information for the base layer image or shapefile. We cannot publish proprietary or copyrighted maps (e.g. Google Maps, Mapquest) and the terms of use for your map base layer must be compatible with our CC BY 4.0 license. Note: if you created the map in a software program like R or ArcGIS, please locate and indicate the source of the basemap shapefile onto which data has been plotted. If your map was obtained from a copyrighted source please amend the figure so that the base map used is from an openly available source. Alternatively, please provide explicit written permission from the copyright holder granting you the right to publish the material under our CC BY 4.0 license. If you are unsure whether you can use a map or not, please do reach out and we will be able to help you. The following websites are good examples of where you can source open access or public domain maps: * U.S. Geological Survey (USGS) - All maps are in the public domain. (http://www.usgs.gov) * PlaniGlobe - All maps are published under a Creative Commons license so please cite u201cPlaniGlobe, http://www.planiglobe.com, CC BY 2.0u201d in the image credit after the caption. (http://www.planiglobe.com/?lang=enl) * Natural Earth - All maps are public domain. (http://www.naturalearthdata.com/about/terms-of-use/).

We look forward to hearing from you in due course.
Kind regards,
Anitha Samidurai
Editorial Office
On behalf of PLOS Neglected Tropical Diseases
