# Supplementary material for: Hepatitis E in suspected acute hepatitis cases in Brazil: A comparison with hepatitis A
Source: PLoS Negl Trop Dis. 2026 Oct 26;20(10):e0014834. doi: 10.1371/journal.pntd.0014834 (PMC13766607; doi:10.1371/journal.pntd.0014834)
Supplement: Supplementary file 4 [file pntd.0014834.s004.docx]

The manuscript has improved substantially compared with the previous version. The reformulation of the study objectives was appropriate and provides better consistency with the analyses and results presented in the manuscript. The statistical analyses are also now presented more clearly and appropriately throughout the manuscript. It was also a good decision to shorten and streamline the Introduction, which is now more focused. The Discussion is also better focused on the results presented in the manuscript.

Some specific comments:

Title. Although the revised title now better reflects the role of hepatitis A in the study, it would be helpful to indicate more explicitly that hepatitis E remains the main focus of the manuscript, particularly given the emphasis on HEV throughout the study and its objectives. Please consider whether the title could be revised accordingly.

Abstract and throughout the manuscript. Please standardize the number of decimal places used when reporting percentages throughout the manuscript and provide the corresponding confidence intervals where appropriate.

Introduction section:

- Page 7. Second paragraph: Please insert a paragraph break between the general descriptions of HEV and HAV. In addition, please revise the phrase “another most enterically transmitted hepatotropic virus,” which is grammatically unclear.
- Page 7. The genomic characteristics of HEV are not described, whereas those of HAV are presented in some detail (e.g., RNA genome, non-enveloped virus). Please consider providing a similar level of basic genomic and structural information for HEV to ensure consistency in the description of both viruses.
- Pages 7–8. The epidemiological distribution of HEV genotypes is mentioned on page 7 and further described on page 8 within the same section of the Introduction. Please consider consolidating this information to avoid redundancy and improve the flow of the text. In addition, the manuscript does not make any reference to HAV genotypes. Please consider whether information on HAV genotypes should also be included.

Methods section:

- Page 11. The first sentence is quite long and contains extensive geographical information, which makes the main point difficult to follow. Please consider shortening or restructuring the sentence to improve clarity and keep the focus on the study population and design.

Discussion

- Figure 2. This figure appears to present results regarding the regional distribution of AHE cases and anti-HEV IgG positivity, but it is currently introduced in the Discussion. Consider moving the figure and its corresponding description to the Results section, where these findings are presented, and referring to it in the Discussion as appropriate. In addition, please check the labeling of panels A and B. The text preceding the figure indicates that panel A shows anti-HEV IgG-positive cases and panel B shows AHE cases, whereas the figure legend indicates the opposite. Please ensure that the panel labels and corresponding descriptions are consistent. Finally, the legend includes a description labeled “(C),” although the figure contains only two panels, and the two panels are not explicitly labeled as A and B in the figure itself. Please revise the figure and legend accordingly.
