# Supplementary material for: Hepatitis E in suspected acute hepatitis cases in Brazil: A comparison with hepatitis A
Source: PLoS Negl Trop Dis. 2026 Oct 26;20(10):e0014834. doi: 10.1371/journal.pntd.0014834 (PMC13766607; doi:10.1371/journal.pntd.0014834)
Supplement: Supplementary file 5 [file pntd.0014834.s005.docx]

# Response to the Editor and Reviewers

**Manuscript ID:** PNTD-D-26-00195R1
**Title:** Clinical and Epidemiological Comparison of Hepatitis A and E Infections in Brazil

Dear Dr. Fantilli, Dr. Seifert, Dr. Kamhavi, Dr. Brindley, and Reviewers,

We sincerely thank you for the careful evaluation of our manuscript and for the constructive comments. We have revised the manuscript accordingly and believe that these changes have improved its clarity, consistency, and presentation. Below, we respond point by point.

## Response to the Additional Editor Comments

**Comment:** The manuscript has been substantially improved in response to the reviewers and editors’ comments. The objectives have been clarified, the structure and presentation of the manuscript have been strengthened, and the statistical analyses and interpretation of the results are now more clearly aligned with the stated aims of the study. At this stage, only minor revisions are required. I encourage the authors to perform a final careful review of the manuscript to ensure consistency in terminology, percentages and decimal places, confidence intervals where applicable and figure references.
In addition, I recommend a final reconsideration of the title. Although the revised title represents an improvement, it could be further refined to more accurately reflect the main objective and scope of the study.

**Response:** We thank the Editor for this recommendation. We carefully reviewed the manuscript and standardized terminology, percentages, decimal places, confidence intervals, and figure citations throughout. We also reconsidered the title and revised it to better reflect the study objective and scope. The revised title is ***Hepatitis E in Suspected Acute Hepatitis Cases in Brazil: A Comparison with Hepatitis A***.

## Response to the Journal Requirements:

**1. Author Summary:** Please provide an Author Summary. This should appear in your manuscript between the Abstract (if applicable) and the Introduction, and should be 150-200 words long. The aim should be to make your findings accessible to a wide audience that includes both scientists and non-scientists. Sample summaries can be found on our website under Submission Guidelines:
https://journals.plos.org/plosntds/s/submission-guidelines#loc-parts-of-a-submission

**Response:** An Author Summary of 150–200 words has been added between the Abstract and the Introduction, in accordance with the journal’s instructions. (page 6)

*“Author summary*

*We conducted a multicenter study of 1,721 adults with suspected acute hepatitis from five Brazilian regions. Anti-HEV IgG was detected in 5.172% of participants, with clear geographic and demographic variation, whereas anti-HAV IgG was much more frequent (78.6%). AHA was much more frequent than AHE: 87.1% vs. 12.9%. We also identified distinct epidemiological profiles: AHE affected older adults, was more frequent among women and heterosexual individuals, and occurred in both urban and rural settings. AHE cases had milder clinical and biochemical profiles than AHA cases, which showed higher aminotransferase levels and more frequent jaundice. These findings highlight an important gap in routine diagnostic testing for acute hepatitis in Brazil, because hepatitis E may be missed without targeted testing. Adding HEV testing to standard diagnostic panels could improve patient care and public health surveillance.”*

**2. Financial Disclosure:** Please amend your detailed Financial Disclosure statement. This is published with the article. It must therefore be completed in full sentences and contain the exact wording you wish to be published.
i) State what role the funders took in the study. If the funders had no role in your study, please state: "The funders had no role in study design, data collection and analysis, decision to publish, or preparation of the manuscript."
ii) If any authors received a salary from any of your funders, please state which authors and which funders..
If you did not receive any funding for this study, please simply state: u201cThe authors received no specific funding for this work.u201d

**Response:** The Financial Disclosure statement has been revised in full sentences and updated in the submission system. The final statement in page 44reads:

*“****Funding***

*This study was supported by Hospital Israelita Albert Einstein through the Program for Supporting the Institutional Development of the Public Health System (PROADI‐SUS), supported by the Ministry of Health of Brazil (NUP:25000.171379/2023-67). The funders had no role in study design, data collection and analysis, decision to publish, or preparation of the manuscript.*

*KGO is a graduate student in the Graduate Program in Gastroenterology Sciences at Faculdade de Medicine, Universidade de São Paulo, NUSP 7069372*

*JRRP receives a fellowship from Conselho Nacional de Desenvolvimento Científico e Tecnológico (CNPq) - Bolsista de produtividade, 306878/2023-2..”*

## Response to Attached Letter sent by the editor

**General comment:** The manuscript has improved substantially compared with the previous version. The reformulation of the study objectives was appropriate and provides better consistency with the analyses and results presented in the manuscript. The statistical analyses are also now presented more clearly and appropriately throughout the manuscript. It was also a good decision to shorten and streamline the Introduction, which is now more focused. The Discussion is also better focused on the results presented in the manuscript.

**Response:** We sincerely thank the Editor for this positive assessment. We appreciate the recognition of the improvements made to the objectives, statistical presentation, Introduction, and Discussion. We have carefully considered the additional specific comments and revised the manuscript, accordingly, as detailed below.

**1. Title**

**Comment:** Although the revised title now better reflects the role of hepatitis A in the study, it would be helpful to indicate more explicitly that hepatitis E remains the main focus of the manuscript, particularly given the emphasis on HEV throughout the study and its objectives. Please consider whether the title could be revised accordingly.

**Response:** As noted in our response to the Additional Editor Comments above, we agree that the title should more explicitly convey the central focus on hepatitis E while retaining the comparative role of hepatitis A. We therefore reconsidered and revised the title accordingly. The revised title is ***Hepatitis E in Suspected Acute Hepatitis Cases in Brazil: A Comparison with Hepatitis A***.

**2. Abstract and throughout the manuscript**

**Comment:** Please standardize the number of decimal places used when reporting percentages throughout the manuscript and provide the corresponding confidence intervals where appropriate.

**Response:** We carefully reviewed the Abstract and the full manuscript and standardized the number of decimal places used for percentages. We also added the corresponding confidence intervals where appropriate and verified consistency across the text, tables, and figures.

**3. Introduction, page 7: separation of the HEV and HAV descriptions**

**Comment:** Please insert a paragraph break between the general descriptions of HEV and HAV. In addition, please revise the phrase “another most enterically transmitted hepatotropic virus,” which is grammatically unclear.

**Response:** We inserted a paragraph break so that the discussion of HAV begins in a separate paragraph. We also revised the grammatically unclear phrase to read.

**Page 8, 1st paragraph:** "HAV is another enterically transmitted hepatotropic virus…”

- **4. Introduction, page 7: genomic and structural characteristics of HEV**

  **Comment:** The genomic characteristics of HEV are not described, whereas those of HAV are presented in some detail (e.g., RNA genome, non-enveloped virus). Please consider providing a similar level of basic genomic and structural information for HEV to ensure consistency in the description of both viruses.

**Response:** We agree and added a concise description of the basic genomic and structural characteristics of HEV to provide a level of detail comparable to that used for HAV and to improve consistency between the descriptions of the two viruses.

**Page 7, 1st paragraph:**

**“Introduction**

HEV was first recognized after large outbreaks of enterically transmitted non-A, non-B hepatitis in Asia, including epidemics in India and Afghanistan, and the viral agent was subsequently visualized as virus-like particles in stool samples [1]. HEV belongs to the family *Hepeviridae*, which includes multiple genera and species with human and animal hosts; among these, *Paslahepevirus balayani* includes the main human HEV genotypes, whereas *Rocahepevirus ratti* includes rat HEV strains with zoonotic potential [2]. *HEV is a small, positive-sense single-stranded RNA virus with a genome of approximately 7.2 kb. The genome contains three principal open reading frames: ORF1 encodes the nonstructural polyprotein* *required for viral replication, ORF2 encodes the capsid protein, and ORF3 encodes a small multifunctional protein involved in virion release and pathogenesis. Virions are non-enveloped in feces but circulate in blood as quasi-enveloped particles surrounded by a host-derived lipid membrane.* [2]”

**5. Introduction, pages 7–8: HEV and HAV genotypes**

**Comment:** The epidemiological distribution of HEV genotypes is mentioned on page 7 and further described on page 8 within the same section of the Introduction. Please consider consolidating this information to avoid redundancy and improve the flow of the text. In addition, the manuscript does not make any reference to HAV genotypes. Please consider whether information on HAV genotypes should also be included.

**Response:** We revised the Introduction to consolidate the information on the epidemiological distribution of HEV genotypes in a single, coherent passage, thereby removing redundancy. We also added a concise statement on HAV genotypes to provide balanced background information on both viruses.

**Introduction, pages 7–8:**

“HEV genotypes differ in host range, geographic distribution, and transmission routes. Genotypes 1 and 2 infect humans and are mainly transmitted through fecally contaminated water, causing large outbreaks in regions with limited sanitation. Genotypes 3 and 4 circulate in animal reservoirs, particularly domestic pigs and wild boars, and cause sporadic zoonotic infections associated with raw or undercooked pork, game meat, shellfish, and other contaminated foods; transfusion-associated transmission has also been reported. Genotypes 5 and 6 have been detected in wild boars, whereas genotypes 7 and 8 occur in dromedaries and Bactrian camels, respectively, with rare human infection linked to camel-derived products [4–8].

HAV is another enterically transmitted hepatotropic virus and remains an important cause of acute viral hepatitis worldwide. HAV is a non-enveloped, positive-sense single-stranded RNA virus of the genus Hepatovirus in the family Picornaviridae. Three genotypes (I–III), each divided into subtypes A and B, infect humans; genotype I is the most prevalent worldwide, with subgenotype IA reported most frequently. HAV is transmitted mainly by the fecal–oral route through contaminated food or water or close person-to-person contact, including oral–anal sexual exposure. Unlike hepatitis B and C viruses, HAV does not cause chronic infection, but disease severity increases with age, and fulminant hepatitis may occur rarely [9–10].”

**6. Methods, page 11**

**Comment:** The first sentence is quite long and contains extensive geographical information, which makes the main point difficult to follow. Please consider shortening or restructuring the sentence to improve clarity and keep the focus on the study population and design.

**Response:** We agree and restructured the opening sentence of the Methods section to improve readability and maintain the focus on the study population and design. The geographical information has been shortened and reorganized without changing the content or scope of the study.

**Methods, page 11:**

“This multicenter prospective observational study sequentially enrolled individuals aged ≥18 years who sought care for clinical signs or symptoms suggestive of acute liver disease between October 2019 and May 2023. Participants were recruited at 31 viral hepatitis referral services across 16 Brazilian states, representing the North, Northeast, South, Southeast, and Central West regions. A total of 1,721 samples were collected. All samples were validated according to the study protocol and had sufficient volume for testing HAV and HEV infection markers.”

**7. Discussion and Figure 2**

**Comment:** This figure appears to present results regarding the regional distribution of AHE cases and anti-HEV IgG positivity, but it is currently introduced in the Discussion. Consider moving the figure and its corresponding description to the Results section, where these findings are presented, and referring to it in the Discussion as appropriate. In addition, please check the labeling of panels A and B. The text preceding the figure indicates that panel A shows anti-HEV IgG-positive cases and panel B shows AHE cases, whereas the figure legend indicates the opposite. Please ensure that the panel labels and corresponding descriptions are consistent. Finally, the legend includes a description labeled “(C),” although the figure contains only two panels, and the two panels are not explicitly labeled as A and B in the figure itself. Please revise the figure and legend accordingly.

**Response:** We agree that Figure 2 presents study findings and therefore belongs in the Results section. We moved Figure 2 and its corresponding descriptive text to the Results section (**page 25**) while retaining the interpretation of the regional findings in the Discussion. We removed the obsolete Figure 2 block from the Discussion, corrected the assignments of panels A and B, and added explicit A and B labels within the figure itself. We also revised the legend so that the panel labels and descriptions are fully consistent. The text describing the map boundaries and figure preparation were changed and the erroneous panel C designation was removed because the figure contains only two panels.

## Methods:

## Response to Reviewer #1

**Comment:** All previous comments were properly addressed.
**Response:** We thank the Reviewer for the positive assessment and for the constructive comments provided during the review process.

## Response to Reviewer #2

**Comment:** The study objectives are clearly articulated and are consistent with the analyses and results presented. The study design and population are appropriate to address the stated objectives, and the statistical analyses are appropriate and adequately support the conclusions. The sample size appears reasonable for the scope and design of the study. I have no concerns regarding ethical or regulatory requirements.

**Response:** We sincerely thank the Reviewer for this positive assessment of the study objectives, design, population, statistical analyses, sample size, and ethical and regulatory aspects. We are pleased that the manuscript is considered methodologically sound and that the analyses adequately support the conclusions. We appreciate the Reviewer’s careful evaluation and constructive contribution to the review process.

## Response to Reviewer #3

**Comment:** The introduction was significantly improved, presenting background information relevant to the paper's purpose and clearly outlining the objectives.
Materials and Methods: Although the case definition for acute hepatitis E has been clarified in accordance with international guidelines, the information presented does not exactly match what some of the guidelines state. In page 13 it is mentioned that acute hepatitis E is defined by the detection of anti-HEV IgM antibodies and/or HEV RNA, noting that all guidelines agree on this point. However, the EASL guideline defines "current acute infection" as the presence of HEV RNA—with or without anti-HEV IgM—but excludes cases where only anti-HEV IgM is present. On the other hand, WHO guidelines do accept the presence of anti-HEV IgM alone, in conjunction with symptoms of acute hepatitis, as a confirmatory diagnostic criterion for the infection. Therefore, the statement should be revised to accurately reflect these differences among the international guidelines.

**Response:** We thank the Reviewer for this important clarification. We revised the Methods to distinguish the guideline definitions accurately. The text now explains that the EASL guideline defines current acute HEV infection by detectable HEV RNA, with or without anti-HEV IgM, whereas the WHO framework accepts anti-HEV IgM in a patient with compatible acute hepatitis as a confirmatory criterion. We no longer state that all guidelines use an identical definition.

**Page 12, last line, and page 13**

“**Case definition of AHE**

AHE was defined using clinical and laboratory criteria informed by international and national recommendations. For this study, a case was classified as AHE when biochemical evidence of acute hepatitis, characterized by a recent elevation in serum aminotransferases, was accompanied by anti-HEV IgM reactivity and/or detectable HEV RNA by nucleic acid amplification testing. The international guidelines differ in the evidentiary weight assigned to these markers. EASL defines current acute HEV infection by detectable HEV RNA, with or without anti-HEV IgM, and does not classify isolated anti-HEV IgM positivity as current acute infection. In contrast, WHO surveillance guidance accepts anti-HEV IgM positivity in a patient with compatible acute hepatitis as laboratory confirmation, while noting that an additional marker, such as HEV RNA, increases diagnostic specificity. Thus, the operational case definition used in this study is consistent with the WHO surveillance approach but is broader than the EASL definition of current acute infection. Isolated anti-HEV IgG positivity was interpreted as evidence of previous exposure and was not used to define acute infection. Participants with anti-HEV IgG reactivity were analyzed separately to assess factors associated with previous HEV exposure. [31–32]”

## Results:

## Response to Reviewer #1

**Comment:** All previous comments were properly addressed.
**Response:** We thank the Reviewer for the positive assessment and for the constructive comments provided during the review process.

## Response to Reviewer #2

**Comment:** The analyses presented are consistent with the stated objectives and analysis plan, and the results are generally clearly and adequately presented. The figures are of sufficient quality for interpretation, although Figure 2 requires revision to ensure consistency between the panel labels, figure legend, and corresponding descriptions.

**Response:** We sincerely thank the Reviewer for this positive assessment of the analyses, presentation of the results, and figure quality. As detailed in our response to the Editor’s Comment 7 (“Discussion and Figure 2”), we revised Figure 2 to ensure full consistency among the panel labels, figure legend, and corresponding descriptions. Specifically, we moved the figure and its descriptive text to the Results section, corrected the assignments of panels A and B, added explicit A and B labels within the figure, and removed the erroneous panel C designation from the legend.

## Response to Reviewer #3

**Comment:** The results are clearly presented. Some revision needed:

Please, remove the sentence “…indicating active infection with low-level viral replication” in page 19, since it is not possible to know the level of viral replication just by the Ct level, moreover in a plasma sample. Regarding this RNA-HEV positive case, did the patient have any history of travel? Additionally, did the patient have any evidence of autoimmune markers? In the reviewer’s response, authors mentioned that the sample was collected 19 days after symptoms onset; please, add this information to the Results section (to the paragraph with the case report). Including this information would add value to the case and all the Results.

**Response:** We thank the reviewer for this important observation and apologize for the previous omission. Because the manuscript focuses primarily on hepatitis-related findings, we did not initially revisit all HIV results available for this case when preparing our earlier response. We removed the statement “indicating active infection with low-level viral replication,” because the Ct value obtained from a qualitative RT-qPCR assay in a plasma sample does not allow a reliable inference regarding the level of viral replication. We also expanded the case description in the Results section, **page 19**. The patient was a 32-year-old man, the plasma sample was collected 19 days after symptom onset, and he denied recent domestic or international travel. Anti-HIV serology was reactive, the HIV RNA level was >5,400,000 IU/mL, and molecular characterization identified HIV-1 group M, subtype B. Autoimmune testing showed low-titer antinuclear antibody reactivity with fine-speckled nuclear and speckled cytoplasmic patterns at 1:80; anti-LKM and ASMA-2 were non-reactive, anti-actin IgG was 38.6, and total IgG was unavailable. The concomitant HIV infection may have contributed to nonspecific autoantibody reactivity through immune dysregulation but not to the direct detection of HEV RNA by RT-qPCR. These findings are now reported descriptively in the revised Results section.

The authors included a Figure (Fig2) in the Discussion section. It is important to note that the maps in this figure present data obtained during the study; therefore, they should be moved to the Results section. These data should then be discussed in the Discussion section, just as the differences observed among the various geographic regions of Brazil were discussed. Please, move the figure to the Results section. Added to this, in the legend of the Figure, there is a point “C” explanation, but there is no panel “C” in the Figure. Please, clarify.

**Response:** We thank the Reviewer for this observation. As detailed above in our response to the Editor’s Comment 7 (“Discussion and Figure 2”) and in our response to Reviewer #2 on the same issue, Figure 2 and its corresponding descriptive text were moved to the Results section (**page 25**), while the interpretation of the regional findings was retained throughout the Discussion. We also corrected the assignments of panels A and B, added explicit panel labels within the figure, revised the legend and corresponding descriptions for consistency, and removed the erroneous panel C designation because the figure contains only two panels.

## Conclusions

## Response to Reviewer #1

**Comment:** All previous comments were properly addressed

**Response:** We sincerely thank the Reviewer for confirming that all previous comments were properly addressed. We appreciate the Reviewer’s careful evaluation and constructive feedback throughout the review process.

## Response to Reviewer #2

**Comment:** The conclusions are supported by the data presented and are consistent with the study objectives and findings. The limitations of the study are adequately described, and the Discussion addresses how the findings contribute to the understanding of hepatitis A and E infections in Brazil.

**Response:** We sincerely thank the Reviewer for this positive assessment. We appreciate the recognition that the conclusions are supported by the data and aligned with the study objectives and findings, that the study limitations are adequately described, and that the Discussion appropriately highlights the contribution of our findings to the understanding of hepatitis A and E infections in Brazil.

## **Editorial and Data Presentation Modifications**

## Response to Reviewer #2

**Comment:** The remaining revisions are mainly editorial and concern clarity, consistency, and presentation of the manuscript. These include minor changes to the text, standardization of data presentation, and revision of a figure.

**Response:** We thank the Reviewer for this assessment. We performed a final editorial review of the manuscript and implemented the requested revisions to improve clarity, consistency, and presentation. Specifically, we revised the relevant passages, standardized the presentation of percentages, decimal places, and confidence intervals where applicable, and corrected Figure 2 and its legend (**page 25**) to ensure consistency among the panel labels, descriptions, and references in the text. Further details of these changes are provided in our responses to the corresponding comments above.

## **Summary and General Comments**

## Response to Reviewer #1

**Comment:** All previous comments were properly addressed and the manuscript has improved significantly

**Response:** We sincerely thank the Reviewer for confirming that all previous comments were properly addressed and that the manuscript has improved significantly. We greatly appreciate the Reviewer’s careful evaluation and constructive feedback throughout the review process.

## Response to Reviewer #2

**Comment:** The manuscript has improved substantially compared with the previous version. The objectives are now clearer and better aligned with the analyses and results, the statistical analyses are more clearly presented, and the Discussion is better focused on the findings. The study addresses a relevant topic and provides useful information on hepatitis A and E infections in Brazil. The remaining comments are mainly related to clarity, consistency, and presentation, and I do not consider that additional experiments or substantial new analyses are required.

**Response:** We sincerely thank the Reviewer for this positive assessment and for recognizing the substantial improvements made to the manuscript. We appreciate the acknowledgment that the objectives, analyses, results, and Discussion are now more clearly aligned and that the study provides relevant information on hepatitis A and E infections in Brazil. In response to the remaining comments, we performed a final careful review and implemented the requested editorial revisions to improve clarity, consistency, and presentation, as detailed in our point-by-point responses above. No additional experiments or substantial analyses were required.

## Response to Reviewer #3

**Comment:**The manuscript has been substantially improved. The objective has been clarified, the different sections have been improved, and even the title has been modified so that it more accurately reflects the work carried out.
The introduction was significantly improved, presenting background information relevant to the paper's purpose and clearly outlining the objectives

**Response:** We sincerely thank the Reviewer for recognizing the substantial improvements made to the manuscript, including the clarification of the study objective, the revision of the title, and the strengthening of the Introduction and other sections. We have carefully considered each of the remaining minor comments and addressed them individually in our point-by-point responses below..
Some minor correction is still needed:
**Comment:**1-Introduction, page 7: when starting to speak of HAV, please, start in a different paragraph.

**Response:** We thank the Reviewer for this suggestion. As detailed above in our response to the Editor’s Comment 3 (“Introduction, page 7: separation of the HEV and HAV descriptions”), we inserted a paragraph break so that the description of HAV now begins in a separate paragraph, in the beginning of **page 8**.

**Comment:**2-Materials and Methods: Although the case definition for acute hepatitis E has been clarified in accordance with international guidelines, the information presented does not exactly match what some of the guidelines state. In page 13 it is mentioned that acute hepatitis E is defined by the detection of anti-HEV IgM antibodies and/or HEV RNA, noting that all guidelines agree on this point. However, the EASL guideline defines "current acute infection" as the presence of HEV RNA—with or without anti-HEV IgM—but excludes cases where only anti-HEV IgM is present. On the other hand, WHO guidelines do accept the presence of anti-HEV IgM alone, in conjunction with symptoms of acute hepatitis, as a confirmatory diagnostic criterion for the infection. Therefore, the statement should be revised to accurately reflect these differences among the international guidelines.

**Response:** We thank the Reviewer for this important clarification. As detailed in our previous response to himself in the Methods section above, we revised the case definition of acute hepatitis E to distinguish accurately between the EASL and WHO criteria. The revised text now states that EASL defines current acute HEV infection by detectable HEV RNA, with or without anti-HEV IgM, whereas WHO accepts anti-HEV IgM in a patient with compatible acute hepatitis as a confirmatory criterion. We also removed the statement that all international guidelines use an identical definition. (**Case definition of AHE, pages 12 and 13**)

**Comment:**3-Resultas Section: Please, remove the sentence “…indicating active infection with low-level viral replication” in page 19, since it is not possible to know the level of viral replication just by the Ct level, moreover in a plasma sample. Regarding this RNA-HEV positive case, did the patient have any history of travel? Additionally, did the patient have any evidence of autoimmune markers? In the reviewer’s response, authors mentioned that the sample was collected 19 days after symptoms onset; please, add this information to the Results section (to the paragraph with the case report). Including this information would add value to the case and all the Results.

**Response:** We thank the Reviewer for this important observation. As detailed in our response to Reviewer #3 in the Results section above, we removed the statement “indicating active infection with low-level viral replication,” because the Ct value obtained from a qualitative RT-qPCR assay in a plasma sample does not permit a reliable inference regarding the level of viral replication. We also expanded the case description to report that the plasma sample was collected 19 days after symptom onset, that the patient denied recent domestic or international travel, and that autoimmune testing showed low-titer antinuclear antibody reactivity, while anti-LKM and ASMA-2 were non-reactive. The additional HIV and autoimmune findings are now described in the revised Results section, **pages 19 and 20**.

**“Demographic, clinical and laboratorial data of the HEV RNA positive case**

The patient was a 32-year-old cisgender man residing in an urban area in Rondônia State, North Brazil. He identified as heterosexual, did not belong to the MSM group, and reported two sexual partners during the previous six months. He had a history of prior surgery (type not specified). The plasma sample was collected 19 days after symptom onset. At enrollment, he reported nausea, fever, and anorexia, without choluria or clinical jaundice, and was classified as anicteric. He reported no seafood consumption. The patient denied recent domestic or international travel.

Biochemical liver tests showed elevated aminotransferases and gamma-glutamyl transferase: AST 224 U/L, ALT 259 U/L, and GGT 259 U/L. Alkaline phosphatase was 99 U/L. Total and fractionated bilirubin levels were normal (total, 0.6 mg/dL; direct, 0.2 mg/dL; indirect, 0.4 mg/dL), consistent with the absence of jaundice. Total IgG was not available.

Laboratory evaluation demonstrated reactive anti-HEV IgM and detectable HEV RNA in plasma by qualitative RT-qPCR (Ct 38.1). Attempts to amplify the target regions for sequencing and genotyping were unsuccessful. HBsAg, total anti-HBc, and anti-HCV were non-reactive. Anti-HIV serology was reactive, with an HIV RNA level >5,400,000 IU/mL; molecular characterization identified HIV-1 group M, subtype B. Anti-HAV IgG was reactive, and HAV RNA was not detected, consistent with previous HAV exposure. Leptospira serology was non-reactive, and toxoplasmosis serology was reactive. Autoimmune testing showed antinuclear antibody reactivity with a fine-speckled nuclear pattern at a titer of 1:80 and a speckled cytoplasmic pattern at 1:80. Nucleolar and mitotic-apparatus patterns were non-reactive. Anti-LKM and ASMA-2 patterns involving the muscularis mucosa, glomerular mesangium, vascular smooth-muscle sheath, and intercellular tubular fibrils were non-reactive. Anti-actin IgG was 38.6 U; however, total IgG was unavailable, and these findings were reported descriptively without establishing autoimmune hepatitis. The concomitant HIV infection may have contributed to nonspecific autoantibody reactivity through immune dysregulation but not to the detection of HEV RNA by RT-qPCR.”

**Comment:**4- The authors included a Figure (Fig2) in the Discussion section. It is important to note that the maps in this figure present data obtained during the study; therefore, they should be moved to the Results section. These data should then be discussed in the Discussion section, just as the differences observed among the various geographic regions of Brazil were discussed. Please, move the figure to the Results section. Added to this, in the legend of the Figure, there is a point “C” explanation, but there is no panel “C” in the Figure. Please, clarify.

**Response:** We thank the Reviewer for this observation. As detailed above in our response to the Editor’s Comment 7 (“Discussion and Figure 2”) and in our response to Reviewer #2 on the same issue, Figure 2 and its corresponding descriptive text were moved to the Results section (**page 25**), while the interpretation of the regional findings was retained trhoughout the Discussion. We also corrected the assignments of panels A and B, added explicit panel labels within the figure, revised the legend and corresponding descriptions for consistency, and removed the erroneous panel C designation because the figure contains only two panels.

**Comment:**5-Discussion section: please, add a sentence mentioning possible reasons for the failure to detect HEV-RNA in patients with acute HEV infection. One possible explanation could be the time elapsed between symptom onset and sample collection (averaging 12 days), given the short duration of viremia. This issue could be addressed by collecting stool samples, as the period of viral excretion is longer (this also could be mentioned).

**Response:** We thank the reviewer for this relevant suggestion. We added a statement to the Discussion addressing possible reasons for the limited detection of HEV RNA among patients classified as having acute hepatitis E. The interval between symptom onset and sample collection had a median of 12.5 days and a mean of 15.75 days. Given the relatively short duration of viremia in acute HEV infection, circulating viral RNA may already have declined below the assay detection limit by the time blood was collected in some patients. We also acknowledge that collecting stool specimens in addition to blood could improve molecular detection, because fecal HEV RNA excretion may persist longer and extend the diagnostic window.

**Page 27:**

“A total of 124 acute infections were identified; 12.9% were identified as AHE and 87.1% as AHA. A review on acute viral hepatitis around the world based on the Global Burden of Disease study 2021 estimated the following number of cases: AHA (160.86 million) and AHE (19.37 million). Projections suggest that by 2030, the global number of AHA cases could decrease to 152.9 million while the global number of AHE cases will rise to 20.5 million.[35] The AHA/AHE proportion we found in Brazil was 6.77 while in the world in 2021 it was 8.30. Other studies addressing AHE should be carried out in our country to further confirm the data shown herein and to better understand the burden of this infection in Brazil. *The limited detection of HEV RNA among patients classified as AHE may partly reflect the interval between symptom onset and sample collection (median, 12.5 days; mean, 15.7 days), given the relatively short duration of viremia in acute HEV infection. By the time blood was collected, circulating viral RNA may already have declined below the assay detection limit in some patients. Future studies should consider collecting stool specimens in addition to blood, because fecal HEV RNA excretion may persist longer and may extend the window for molecular detection.”*

We again thank the Editors and Reviewers for their thoughtful comments, which have helped us improve the manuscript. We hope that the revised version is now suitable for publication in *PLOS Neglected Tropical Diseases*.

Sincerely,

João Renato Rebello Pinho
on behalf of all authors
