# Supplementary material for: Hepatitis E in suspected acute hepatitis cases in Brazil: A comparison with hepatitis A
Source: PLoS Negl Trop Dis. 2026 Oct 26;20(10):e0014834. doi: 10.1371/journal.pntd.0014834 (PMC13766607; doi:10.1371/journal.pntd.0014834)
Supplement: Peer review history

# Peer review: Decision Letter 0

**DOI:** 10.1371/journal.pntd.0014834.r001

**Authors:** Anabella Clara Fantilli, Stephanie N. Seifert

5 May 2026

Hepatitis E in Brazil: Investigation of Suspected Acute Hepatitis Cases

PLOS Neglected Tropical Diseases

Dear Dr. Pinho,

Thank you for submitting your manuscript to PLOS Neglected Tropical Diseases. After careful consideration, we feel that it has merit but does not fully meet PLOS Neglected Tropical Diseases's publication criteria as it currently stands. Therefore, we invite you to submit a revised version of the manuscript that addresses the points raised during the review process.

Please submit your revised manuscript within by Jul 04 2026 11:59PM. If you will need more time than this to complete your revisions, please reply to this message or contact the journal office at plosntds@plos.org. When you're ready to submit your revision, log on to https://www.editorialmanager.com/pntd/ and select the 'Submissions Needing Revision' folder to locate your manuscript file.

Please include the following items when submitting your revised manuscript:

* A letter that responds to each point raised by the editor and reviewer(s). You should upload this letter as a separate file labeled 'Response to Reviewers'. This file does not need to include responses to any formatting updates and technical items listed in the 'Journal Requirements' section below.

* A marked-up copy of your manuscript that highlights changes made to the original version. You should upload this as a separate file labeled 'Revised Manuscript with Track Changes'.

* An unmarked version of your revised paper without tracked changes. You should upload this as a separate file labeled 'Manuscript'.

If you would like to make changes to your financial disclosure, competing interests statement, or data availability statement, please make these updates within the submission form at the time of resubmission. Guidelines for resubmitting your figure files are available below the reviewer comments at the end of this letter.

We look forward to receiving your revised manuscript.

Kind regards,

Anabella Clara Fantilli

Guest Editor

PLOS Neglected Tropical Diseases

Stephanie Seifert

Section Editor

PLOS Neglected Tropical Diseases

Shaden Kamhawi

co-Editor-in-Chief

PLOS Neglected Tropical Diseases

orcid.org/0000-0003-4304-636XX

Paul Brindley

co-Editor-in-Chief

PLOS Neglected Tropical Diseases

orcid.org/0000-0003-1765-0002

**Additional Editor Comments:**

Overall, there are several important issues that need to be addressed before a final decision can be made. A major concern raised consistently by all reviewers relates to the need for greater clarity and consistency across the manuscript, particularly regarding the study objectives and their alignment with the Methods and Results sections. In its current form, there is some ambiguity about the role of HAV in the study (i.e., whether it is part of the main objective or primarily included for comparative purposes with HEV).

In addition, the Methods section requires further detail and clarification, most importantly regarding the criteria used for the diagnosis of acute hepatitis E, which is essential for the interpretation of the results.The Results section would benefit from the inclusion of currently missing or insufficiently reported data, and the Discussion and Conclusion requires further refinement to ensure that interpretations are fully supported by the results presented and that certain statements are appropriately qualified.

Beyond the reviewers’ suggestions, I would like to highlight a few additional points. First, the manuscript would benefit from a thorough revision of the English language, as there are several grammatical issues and redundancies throughout the text (e.g., repeated definitions of abbreviations such as HEV, AHA, AHE, MSM). Improving the overall readability will greatly strengthen the manuscript.

Second, regarding the previous seroprevalence data mentioned in the Introduction and Discussion, it is important to acknowledge that part of the heterogeneity observed across regions may be due to the use of different serological assays in previous studies, which complicates direct comparisons. This aspect should be explicitly considered when interpreting and discussing regional differences. In this context, a strength of the present study is that it provides more comparable seroprevalence estimates across different regions, as the same assay was consistently used for all analyzed samples.

Finally, please ensure that all requested revisions are carefully addressed, as well as any additional issues identified during your revision. A final decision will be made based on the revised version of the manuscript.

**Journal Requirements:**

1) Please ensure that the CRediT author contributions listed for every co-author are completed accurately and in full.

At this stage, the following Authors/Authors require contributions: Ana Catharina de Seixas Santos Nastri, and Mario Peribañez Gonzalez. Please ensure that the full contributions of each author are acknowledged in the "Add/Edit/Remove Authors" section of our submission form.

The list of CRediT author contributions may be found here: https://journals.plos.org/plosntds/s/authorship#loc-author-contributions

2) We do not publish any copyright or trademark symbols that usually accompany proprietary names, eg ©,  ®, or TM  (e.g. next to drug or reagent names). Therefore please remove all instances of trademark/copyright symbols throughout the text, including:

- ® on page: 13.

3) Please upload all main figures as separate Figure files in .tif or .eps format. For more information about how to convert and format your figure files please see our guidelines:

https://journals.plos.org/plosntds/s/figures

4) Please amend your detailed Financial Disclosure statement. This is published with the article. It must therefore be completed in full sentences and contain the exact wording you wish to be published.

- State the initials, alongside each funding source, of each author to receive each grant. For example: "This work was supported by the National Institutes of Health (####### to AM; ###### to CJ) and the National Science Foundation (###### to AM)."

- State what role the funders took in the study. If the funders had no role in your study, please state: "The funders had no role in study design, data collection and analysis, decision to publish, or preparation of the manuscript.".

If you did not receive any funding for this study, please simply state: u201cThe authors received no specific funding for this work.u201d

**Reviewers' Comments:**

Reviewer's Responses to Questions

**Key Review Criteria Required for Acceptance?**

As you describe the new analyses required for acceptance, please consider the following:

**Methods**

-Are the objectives of the study clearly articulated with a clear testable hypothesis stated?

-Is the study design appropriate to address the stated objectives?

-Is the population clearly described and appropriate for the hypothesis being tested?

-Is the sample size sufficient to ensure adequate power to address the hypothesis being tested?

-Were correct statistical analysis used to support conclusions?

-Are there concerns about ethical or regulatory requirements being met?

Reviewer #1: 1. Authors refer to a previous published work where samples were collected, however some specifications should be included in this manuscript as well; e.g. number of samples included, the inclusion criteria should be described in more detailed (hematological, biochemical parameters, tests for other hepatitis virus or associated viruses).

2. HAV vaccination should be more explicitly mentioned and treated as a variable in the study, also it would be important to describe and discuss the HAV vaccination program and coverage throughout Brazil.

3. Regarding the only RT-qPCR positive sample:

a. What was the Ct and RNA concentration obtained?

b. What epidemiological and socio-demographic characteristics presented this patient?

c. When authors mention that this sample could not be sequenced, which PCR method was employed? Was it due to low viral titer or lack of sensitivity of the test selected? Please clarify this point in the manuscript.

Ethical comitee approval should be explicitly included in this manuscript, not only cited the previous work where the samples were obtained.

Reviewer #2: The objectives of the study are relevant; however, there is an inconsistency between how they are presented in the Abstract and in the main manuscript. In particular, the relationship between the different analytical components described in the Methods and the stated objectives is not entirely clear, which may lead to some confusion regarding the scope of the study. The multicenter prospective design is appropriate to address the study, and the study population is clearly described and suitable, including adults with suspected acute hepatitis across multiple regions of Brazil. The overall sample size is large and adequate for the main analyses. The statistical methods used are appropriate and adequately described; however, more complete reporting of the variables analyzed, including those not found to be statistically significant (see specific comment), would improve transparency.

Reviewer #3: The title does not mention the detection of HAV. Please, modify it including HAV.

In the Abstract, it is mentioned that anti‑HEV IgG prevalence was higher among men who have sex with men (MSM), whereas in the text, it is stated that the association is with heterosexual persons. Please, clarify.

1-The introduction is too long. While the information provided is relevant to both the topic and the article, it is presented with an excessive level of detail which is irrelevant to the article's objective. For instance, the paragraph 2 of page 11, enumerates all reports of HEV infections with viral RNA detection, which is unnecessarily exhaustive for a paper’s introduction. A more concise approach—briefly acknowledging the existence of prior reports and summarizing key aspects such as detected genotypes/subtypes, studied populations, and types of biological samples—would be sufficient to contextualize the study. The same applies to the second paragraph on page 10 of the introduction, which mentions studies conducted between 1999 and 2011. Please revise this section accordingly, ensuring that both these paragraphs and the entire introduction are appropriately summarized.

2-Remove the following sentence (introduction, page 10), as rat HEV (Rocahepevirus ratti) is a different virus and this study focuses on HEV (Paslahepevirus balayani): “In bivalve mollusks sampled in Maranhão state, rat HEV RNA was detected in 2.2% of samples [17].”

3-Introduction, page 9, second paragraph, line 5: add the word “than” between “more” and “2 weeks”, so the sentence says “more than 2 weeks”.

4- Although the stated objective of the study is “to determine the prevalence of HEV infection through serological analysis of anti-HEV IgG and IgM antibodies, as well as molecular detection of viral RNA”, it appears that the actual aim is to determine whether HEV is a causative agent of acute infections in Brazil and at what frequency. Please clarify this point in the Introduction.

**********

**Results**

-Does the analysis presented match the analysis plan?

-Are the results clearly and completely presented?

-Are the figures (Tables, Images) of sufficient quality for clarity?

Reviewer #1: 4. I suggest authors to include a map of Brazil showing the distribution of the detected HEV cases for a more graphical visualization.

5. The Discussion section lacks information about the selected sampled regions, such as its different socio-demographic and geographic contexts or other relevant aspects that could help enrich the discussion as a possible association with HEV cases.

Reviewer #2: The analyses presented are generally consistent with the stated analysis plan, including both the comparison between acute hepatitis A and E cases and the evaluation of factors associated with anti-HEV IgG seropositivity. The results are clearly presented overall and follow a logical structure; however, in some instances, only statistically significant findings are reported. The tables are of adequate quality and generally clear.

Reviewer #3: 5-In the first paragraph of the results section (page 15), it is stated that “16 patients were classified as acute hepatitis E, all of them anti-HEV IgM (+) but just one with detectable HEV RNA by PCR”. Regarding this, what criteria were used for the diagnosis of acute hepatitis E, considering that there are discrepancies worldwide? For example, the European Association for the Study of the Liver (EASL) guidelines state that the diagnosis of acute hepatitis E is established by the presence of HEV-RNA with or without serological markers (specific IgM and/or IgG), or by the presence of IgM together with rising anti-HEV IgG levels. Haw was the specific IgG status in the IgM+ patients? The criterion used is crucial and must be clarified in the introduction or materials and methods sections.

6-Regarding the sentence (page 16, Results section) “Anti-HEV IgG antibodies, indicating previous exposure to hepatitis E virus, were present in 5.17% (n = 89) of the study population, while anti-HAV IgG antibodies were observed in a substantially higher proportion of participants (78.56%; n = 1,352)”: Were any of the anti-HEV IgG–positive samples from the same patients who also had detectable anti-HEV IgM? The same clarification applies to HAV. Please clarify this point, as in such cases these would not represent past infections, but rather the presence of an additional marker in the context of an acute infection, and the seroprevalence percentages could therefore vary.

7-Table 1: there is an extra parenthesis (first line). The p value shown, belongs to HAV or HEV? This is not clear in the tables, please clarify there.

8-It is noteworthy that antibodies against HEV (both IgM and IgG) are related to the presence of anti-Toxoplasma IgG. Could this be a cross-reactivity? What are the reactivity levels of the anti-HEV antibodies? This might provide a clue.

9-Only one sample tested positive for HEV RNA; how many days after symptom onset was this patient at the time of sampling? What about those who were HEV IgM-positive? What were the antibody reactivity indices for the detected cases? Cross-reactivity between HEV and Epstein-Barr virus or cytomegalovirus has been reported (see references below), could this be a possibility here? How were the serological markers for this virus?

References:

Fogeda et al. 2009. J Clin Virol. 45 (3): 259-261. https://doi.org/10.1016/j.jcv.2009.05.022

Hyams et al. 2013. Med Virol. https://doi.org/10.1002/jmv.23827

10-In the Discussion section, it is stated that “sequencing of the single HEV RNA–positive sample was not possible, precluding direct characterization of the circulating genotypes. Nonetheless, the predominantly milder clinical presentations observed, together with lower and short-lived viral loads, agree with findings from previous studies conducted in Brazil…”. However, although milder clinical symptoms and low viral loads are mentioned here, these data are not presented in the Results section. If these analyses were performed, please include these correlations in the Results section (i.e., clinical symptoms of patients with hepatitis E and the corresponding viral load measurements).

11-In the last paragraph of page 23, where it states “exhibited significantly lower odds of IgG seropositivity,” please specify “HEV IgG.”

12-In the Discussion section (pages 24 and 25), it is stated: “Indeed, there is no strong evidence that MSM have lower HEV frequency and there is some suggestion of possible sexual transmission in MSM, but there are also multiple studies with no association. Finally, we agree that more studies should be carried out and for the moment, despite conflicting data, hepatitis E may be sexually transmissible in MSM, and MSM with unexplained jaundice, increased transaminases, renal disease or neurological disease should be tested.” It is important to clarify at this point that HEV is not considered a sexually transmitted virus, similar to HAV. Rather, these viruses—particularly HAV, for which numerous reports exist worldwide—are associated with sexual practices involving fecal–oral exposure (e.g., hand–anus–mouth contact), rather than transmission through mucosal sexual contact. Therefore, referring to these cases as “sexual transmission” may be misleading. Please, remove this term and instead clarify this distinction.

13-In the Discussion (page 25), it is stated that “Clinically, AHA was associated with symptomatic presentations with jaundice (86.1%), as well as with asymptomatic or non-jaundiced symptomatic cases (9.3% and 4.6%, respectively). In contrast, all AHE cases were symptomatic, with 56.2% presenting without jaundice and 43.8% with jaundice”, also “Although jaundice and choluria were less frequent in AHE vs. AHA (43.8% vs. 86.1% and 56.3% vs. 83.3%), clinical pictures are similar…”, and “The markedly higher aminotransferase levels in the AHA group (ALT 1,150 U/L; AST 599 U/L) compared with the AHE group (ALT 233 U/L; AST 113 U/L) are in keeping with the typical biochemical profile of acute hepatitis A, which often causes abrupt and pronounced hepatocellular injury with very large transaminase elevations”. However, these data are not presented in the Results section (or briefly mentioned). Please include them there if they are to be discussed.

14-Regarding the sentence “Nevertheless, prior population-based studies in asymptomatic individuals have demonstrated anti-HEV IgM positivity [24,39], supporting the notion of subclinical circulation of the virus and highlighting the potential underestimation of true HEV seroprevalence” of the Discussion section (page 25), were any of the HEV IgM–positive samples obtained from asymptomatic individuals, or did they all correspond to symptomatic patients? This is an important point that should be reported in the Results section and potentially addressed in the Discussion.

**********

**Conclusions**

-Are the conclusions supported by the data presented?

-Are the limitations of analysis clearly described?

-Do the authors discuss how these data can be helpful to advance our understanding of the topic under study?

-Is public health relevance addressed?

Reviewer #1: The limitations of the study are not clearly presented in the conclusions

Reviewer #2: The conclusions are supported by the data presented and are consistent with the main findings of the study, particularly regarding regional differences, age-related trends, and the proportion of acute hepatitis attributable to HEV. The comparison between HAV and HEV is appropriately highlighted and contributes to the understanding of their distinct epidemiological patterns. The manuscript does effectively discuss the relevance of the findings in advancing the understanding of HEV epidemiology, and the public health implications are clearly stated, especially in terms of surveillance and diagnostic strategies.

Reviewer #3: 15-Conclusions: Before mentioning that “HEV accounted for 12.9% of enterically transmitted acute viral hepatitis cases”, the diagnostic criteria for acute hepatitis E should be reconsidered, taking into account international guidelines (such as those from the EASL previously mentioned or the European Centre for Disease Prevention and Control). There is also likely a diagnostic criterion (with its corresponding algorithm) from the Brazilian Ministry of Health, which should be referenced somewhere in the manuscript.

**********

**Editorial and Data Presentation Modifications?**

Use this section for editorial suggestions as well as relatively minor modifications of existing data that would enhance clarity. If the only modifications needed are minor and/or editorial, you may wish to recommend “Minor Revision” or “Accept”.

Reviewer #1: (No Response)

Reviewer #2: Minor specific comments:

Abstract:

A minor wording adjustment in the Abstract objective may help clarify the role of HAV as a reference group.

Please specify whether acute infections were identified by detection of RNA, IgM, or both.

Objective - page 12:

“Patients with suspected acute hepatitis were obtained from …” should be revised, as this wording is not standard for study populations. Consider using “… were enrolled” or “… were included.”

Results – page 16

Table 2 includes only statistically significant associations. It would be helpful to also provide information on the variables analyzed that were not significantly associated, either in the table or as supplementary material, to give a more complete overview of the analysis.

Discussion – page 27:

It may be helpful to briefly mention the hepatitis A vaccination program in Brazil, as this may have influenced the observed anti-HAV IgG seroprevalence and its comparison with HEV.

Reviewer #3: (No Response)

**********

**Summary and General Comments**

Use this section to provide overall comments, discuss strengths/weaknesses of the study, novelty, significance, general execution and scholarship. You may also include additional comments for the author, including concerns about dual publication, research ethics, or publication ethics. If requesting major revision, please articulate the new experiments that are needed.

Reviewer #1: The manuscript "Hepatitis E in Brazil: Investigation of Suspected Acute Hepatitis Cases" reports a multicenter study of hepatitis E in patients with acute hepatitis from Brazil, correlated to hepatitis A cases and socio-demographic factors, which could help expand the epidemiological knowledge of this disease in Brazil.

The manuscript is generally well-written, but some points in the methodology need to be further described.

Reviewer #2: This manuscript addresses a relevant and underrecognized topic and provides valuable data on HEV epidemiology in Brazil. Overall, the study is well conducted and the findings are of interest for both clinical and public health perspectives. A key point for improvement relates to the consistency and clarity of the study objectives across sections, which would benefit from better alignment with the analytical approach described in the Methods. Addressing this aspect, along with the additional comments outlined above, would strengthen the work.

Reviewer #3: This article provides valuable information on HEV and the acute infections it causes in the Brazilian population, covering broad regions of the country and analyzing a large number of samples. However, major revisions are required before it can be published (see comments for each section).

**********

PLOS authors have the option to publish the peer review history of their article (what does this mean?). If published, this will include your full peer review and any attached files.

If you choose “no”, your identity will remain anonymous but your review may still be made public.

**Do you want your identity to be public for this peer review?**  For information about this choice, including consent withdrawal, please see our Privacy Policy.

Reviewer #1: No

Reviewer #2: No

Reviewer #3: No

[NOTE: If reviewer comments were submitted as an attachment file, they will be attached to this email and accessible via the submission site. Please log into your account, locate the manuscript record, and check for the action link "View Attachments". If this link does not appear, there are no attachment files.]

**Figure resubmission:**

**Reproducibility:**

To enhance the reproducibility of your results, we recommend that authors of applicable studies deposit laboratory protocols in protocols.io, where a protocol can be assigned its own identifier (DOI) such that it can be cited independently in the future. Additionally, PLOS ONE offers an option to publish peer-reviewed clinical study protocols. Read more information on sharing protocols at https://plos.org/protocols?utm_medium=editorial-email&utm_source=authorletters&utm_campaign=protocols

# Author comment: Author response to Decision Letter 1

**DOI:** 10.1371/journal.pntd.0014834.r002

24 Aug 2026

Attachment Submitted filename: Response Letter.docx

# Peer review: Decision Letter 1

**DOI:** 10.1371/journal.pntd.0014834.r003

**Authors:** Anabella Clara Fantilli, Stephanie N. Seifert

5 Oct 2026

Clinical and Epidemiological Comparison of Hepatitis A and E Infections in Brazil

Response to ReviewersRevised Manuscript with Track ChangesManuscript'.

Shaden Kamhawi

co-Editor-in-Chief

PLOS Neglected Tropical Diseases

orcid.org/0000-0003-4304-636XX

Paul Brindley

co-Editor-in-Chief

PLOS Neglected Tropical Diseases

orcid.org/0000-0003-1765-0002

**Additional Editor Comments (if provided):**

**Journal Requirements:**

1) Please provide an Author Summary. This should appear in your manuscript between the Abstract (if applicable) and the Introduction, and should be 150-200 words long. The aim should be to make your findings accessible to a wide audience that includes both scientists and non-scientists. Sample summaries can be found on our website under Submission Guidelines:

https://journals.plos.org/plosntds/s/submission-guidelines#loc-parts-of-a-submission

2) Please amend your detailed Financial Disclosure statement. This is published with the article. It must therefore be completed in full sentences and contain the exact wording you wish to be published.

i) State what role the funders took in the study. If the funders had no role in your study, please state: "The funders had no role in study design, data collection and analysis, decision to publish, or preparation of the manuscript."

ii) If any authors received a salary from any of your funders, please state which authors and which funders..

If you did not receive any funding for this study, please simply state: u201cThe authors received no specific funding for this work.u201d

**Reviewers' comments:**

**Key Review Criteria Required for Acceptance?**

As you describe the new analyses required for acceptance, please consider the following:

**Methods**

-Are the objectives of the study clearly articulated with a clear testable hypothesis stated?

-Is the study design appropriate to address the stated objectives?

-Is the population clearly described and appropriate for the hypothesis being tested?

-Is the sample size sufficient to ensure adequate power to address the hypothesis being tested?

-Were correct statistical analysis used to support conclusions?

-Are there concerns about ethical or regulatory requirements being met?

Reviewer #1: All previous comments were properly addressed

Reviewer #2: The study objectives are clearly articulated and are consistent with the analyses and results presented. The study design and population are appropriate to address the stated objectives, and the statistical analyses are appropriate and adequately support the conclusions. The sample size appears reasonable for the scope and design of the study. I have no concerns regarding ethical or regulatory requirements.

Reviewer #3: The introduction was significantly improved, presenting background information relevant to the paper's purpose and clearly outlining the objectives.

Materials and Methods: Although the case definition for acute hepatitis E has been clarified in accordance with international guidelines, the information presented does not exactly match what some of the guidelines state. In page 13 it is mentioned that acute hepatitis E is defined by the detection of anti-HEV IgM antibodies and/or HEV RNA, noting that all guidelines agree on this point. However, the EASL guideline defines "current acute infection" as the presence of HEV RNA—with or without anti-HEV IgM—but excludes cases where only anti-HEV IgM is present. On the other hand, WHO guidelines do accept the presence of anti-HEV IgM alone, in conjunction with symptoms of acute hepatitis, as a confirmatory diagnostic criterion for the infection. Therefore, the statement should be revised to accurately reflect these differences among the international guidelines.

**********

**Results**

-Does the analysis presented match the analysis plan?

-Are the results clearly and completely presented?

-Are the figures (Tables, Images) of sufficient quality for clarity?

Reviewer #1: All previous comments were properly addressed

Reviewer #2: The analyses presented are consistent with the stated objectives and analysis plan, and the results are generally clearly and adequately presented. The figures are of sufficient quality for interpretation, although Figure 2 requires revision to ensure consistency between the panel labels, figure legend, and corresponding descriptions.

Reviewer #3: The results are clearly presented. Some revision needed:

Please, remove the sentence “…indicating active infection with low-level viral replication” in page 19, since it is not possible to know the level of viral replication just by the Ct level, moreover in a plasma sample. Regarding this RNA-HEV positive case, did the patient have any history of travel? Additionally, did the patient have any evidence of autoimmune markers? In the reviewer’s response, authors mentioned that the sample was collected 19 days after symptoms onset; please, add this information to the Results section (to the paragraph with the case report). Including this information would add value to the case and all the Results.

The authors included a Figure (Fig2) in the Discussion section. It is important to note that the maps in this figure present data obtained during the study; therefore, they should be moved to the Results section. These data should then be discussed in the Discussion section, just as the differences observed among the various geographic regions of Brazil were discussed. Please, move the figure to the Results section. Added to this, in the legend of the Figure, there is a point “C” explanation, but there is no panel “C” in the Figure. Please, clarify.

**********

**Conclusions**

-Are the conclusions supported by the data presented?

-Are the limitations of analysis clearly described?

-Do the authors discuss how these data can be helpful to advance our understanding of the topic under study?

-Is public health relevance addressed?

Reviewer #1: All previous comments were properly addressed

Reviewer #2: The conclusions are supported by the data presented and are consistent with the study objectives and findings. The limitations of the study are adequately described, and the Discussion addresses how the findings contribute to the understanding of hepatitis A and E infections in Brazil.

Reviewer #3: (No Response)

**********

**Editorial and Data Presentation Modifications?**

Use this section for editorial suggestions as well as relatively minor modifications of existing data that would enhance clarity. If the only modifications needed are minor and/or editorial, you may wish to recommend “Minor Revision” or “Accept”.

Reviewer #1: (No Response)

Reviewer #2: The remaining revisions are mainly editorial and concern clarity, consistency, and presentation of the manuscript. These include minor changes to the text, standardization of data presentation, and revision of a figure.

Reviewer #3: (No Response)

**********

**Summary and General Comments**

Use this section to provide overall comments, discuss strengths/weaknesses of the study, novelty, significance, general execution and scholarship. You may also include additional comments for the author, including concerns about dual publication, research ethics, or publication ethics. If requesting major revision, please articulate the new experiments that are needed.

Reviewer #1: All previous comments were properly addressed and the manuscript has improved significantly

Reviewer #2: The manuscript has improved substantially compared with the previous version. The objectives are now clearer and better aligned with the analyses and results, the statistical analyses are more clearly presented, and the Discussion is better focused on the findings. The study addresses a relevant topic and provides useful information on hepatitis A and E infections in Brazil. The remaining comments are mainly related to clarity, consistency, and presentation, and I do not consider that additional experiments or substantial new analyses are required.

Reviewer #3: The manuscript has been substantially improved. The objective has been clarified, the different sections have been improved, and even the title has been modified so that it more accurately reflects the work carried out.

The introduction was significantly improved, presenting background information relevant to the paper's purpose and clearly outlining the objectives.

Some minor correction is still needed:

1-Introduction, page 7: when starting to speak of HAV, please, start in a different paragraph.

2-Materials and Methods: Although the case definition for acute hepatitis E has been clarified in accordance with international guidelines, the information presented does not exactly match what some of the guidelines state. In page 13 it is mentioned that acute hepatitis E is defined by the detection of anti-HEV IgM antibodies and/or HEV RNA, noting that all guidelines agree on this point. However, the EASL guideline defines "current acute infection" as the presence of HEV RNA—with or without anti-HEV IgM—but excludes cases where only anti-HEV IgM is present. On the other hand, WHO guidelines do accept the presence of anti-HEV IgM alone, in conjunction with symptoms of acute hepatitis, as a confirmatory diagnostic criterion for the infection. Therefore, the statement should be revised to accurately reflect these differences among the international guidelines.

3-Resultas Section: Please, remove the sentence “…indicating active infection with low-level viral replication” in page 19, since it is not possible to know the level of viral replication just by the Ct level, moreover in a plasma sample. Regarding this RNA-HEV positive case, did the patient have any history of travel? Additionally, did the patient have any evidence of autoimmune markers? In the reviewer’s response, authors mentioned that the sample was collected 19 days after symptoms onset; please, add this information to the Results section (to the paragraph with the case report). Including this information would add value to the case and all the Results.

4- The authors included a Figure (Fig2) in the Discussion section. It is important to note that the maps in this figure present data obtained during the study; therefore, they should be moved to the Results section. These data should then be discussed in the Discussion section, just as the differences observed among the various geographic regions of Brazil were discussed. Please, move the figure to the Results section. Added to this, in the legend of the Figure, there is a point “C” explanation, but there is no panel “C” in the Figure. Please, clarify.

5-Discussion section: please, add a sentence mentioning possible reasons for the failure to detect HEV-RNA in patients with acute HEV infection. One possible explanation could be the time elapsed between symptom onset and sample collection (averaging 12 days), given the short duration of viremia. This issue could be addressed by collecting stool samples, as the period of viral excretion is longer (this also could be mentioned).

**********

PLOS authors have the option to publish the peer review history of their article (what does this mean?). If published, this will include your full peer review and any attached files.

If you choose “no”, your identity will remain anonymous but your review may still be made public.

**Do you want your identity to be public for this peer review?**  For information about this choice, including consent withdrawal, please see our Privacy Policy.

Reviewer #1: No

Reviewer #2: No

Reviewer #3: No

**Figure resubmission:**

**Reproducibility:** -->To enhance the reproducibility of your results, we recommend that authors of applicable studies deposit laboratory protocols in protocols.io, where a protocol can be assigned its own identifier (DOI) such that it can be cited independently in the future. Additionally, PLOS ONE offers an option to publish peer-reviewed clinical study protocols. Read more information on sharing protocols at https://plos.org/protocols?utm_medium=editorial-email&utm_source=authorletters&utm_campaign=protocols-->?>

Attachment Submitted filename: R1.docx

# Author comment: Author response to Decision Letter 2

**DOI:** 10.1371/journal.pntd.0014834.r004

10 Oct 2026

Attachment Submitted filename: Response to Reviewers2.docx

# Peer review: Decision Letter 2

**DOI:** 10.1371/journal.pntd.0014834.r005

**Authors:** Anabella Clara Fantilli, Stephanie N. Seifert

19 Oct 2026

Dear Dr Pinho,

We are pleased to inform you that your manuscript 'Hepatitis E in Suspected Acute Hepatitis Cases in Brazil: A Comparison with Hepatitis A' has been provisionally accepted for publication in PLOS Neglected Tropical Diseases.

Before your manuscript can be formally accepted you will need to complete some formatting changes, which you will receive in a follow up email. A member of our team will be in touch with a set of requests.

Please note that your manuscript will not be scheduled for publication until you have made the required changes, so a swift response is appreciated.

IMPORTANT: The editorial review process is now complete. PLOS will only permit corrections to spelling, formatting or significant scientific errors from this point onwards. Requests for major changes, or any which affect the scientific understanding of your work, will cause delays to the publication date of your manuscript.

Should you, your institution's press office or the journal office choose to press release your paper, you will automatically be opted out of early publication. We ask that you notify us now if you or your institution is planning to press release the article. All press must be co-ordinated with PLOS.

Thank you again for supporting Open Access publishing; we are looking forward to publishing your work in PLOS Neglected Tropical Diseases.

Best regards,

Anabella Clara Fantilli

Guest Editor

PLOS Neglected Tropical Diseases

Stephanie Seifert

Section Editor

PLOS Neglected Tropical Diseases

Shaden Kamhawi

co-Editor-in-Chief

PLOS Neglected Tropical Diseases

orcid.org/0000-0003-4304-636XX

Paul Brindley

co-Editor-in-Chief

PLOS Neglected Tropical Diseases

orcid.org/0000-0003-1765-0002

***********************************************************

p.p1 {margin: 0.0px 0.0px 0.0px 0.0px; line-height: 16.0px; font: 14.0px Arial; color: #323333; -webkit-text-stroke: #323333}span.s1 {font-kerning: none

Reviewer's Responses to Questions

**Key Review Criteria Required for Acceptance?**

As you describe the new analyses required for acceptance, please consider the following:

**Methods**

-Are the objectives of the study clearly articulated with a clear testable hypothesis stated?

-Is the study design appropriate to address the stated objectives?

-Is the population clearly described and appropriate for the hypothesis being tested?

-Is the sample size sufficient to ensure adequate power to address the hypothesis being tested?

-Were correct statistical analysis used to support conclusions?

-Are there concerns about ethical or regulatory requirements being met?

Reviewer #2: (No Response)

Reviewer #3: (No Response)

**********

**Results**

-Does the analysis presented match the analysis plan?

-Are the results clearly and completely presented?

-Are the figures (Tables, Images) of sufficient quality for clarity?

Reviewer #2: (No Response)

Reviewer #3: (No Response)

**********

**Conclusions**

-Are the conclusions supported by the data presented?

-Are the limitations of analysis clearly described?

-Do the authors discuss how these data can be helpful to advance our understanding of the topic under study?

-Is public health relevance addressed?

Reviewer #2: (No Response)

Reviewer #3: (No Response)

**********

**Editorial and Data Presentation Modifications?**

Use this section for editorial suggestions as well as relatively minor modifications of existing data that would enhance clarity. If the only modifications needed are minor and/or editorial, you may wish to recommend “Minor Revision” or “Accept”.

Reviewer #2: (No Response)

Reviewer #3: (No Response)

**********

**Summary and General Comments**

Use this section to provide overall comments, discuss strengths/weaknesses of the study, novelty, significance, general execution and scholarship. You may also include additional comments for the author, including concerns about dual publication, research ethics, or publication ethics. If requesting major revision, please articulate the new experiments that are needed.

Reviewer #2: I thank the authors for their careful revisions. The previous comments have been adequately addressed: the title now better reflects the focus on hepatitis E, the introduction has been improved with a clearer separation of the HEV and HAV descriptions, figure 2 has been moved to the results section with corrected panel labels and legend, and the discussion now addresses the possible reasons for the limited detection of HEV RNA.

Reviewer #3: All requested changes and suggestions were implemented; the article improved significantly.

**********

PLOS authors have the option to publish the peer review history of their article (what does this mean?). If published, this will include your full peer review and any attached files.

If you choose “no”, your identity will remain anonymous but your review may still be made public.

**Do you want your identity to be public for this peer review?**  For information about this choice, including consent withdrawal, please see our Privacy Policy.

Reviewer #2: No

Reviewer #3: No

# Editor report: Acceptance letter

**DOI:** 10.1371/journal.pntd.0014834.r006

**Authors:** Anabella Clara Fantilli, Stephanie N. Seifert

Dear Dr Pinho,

We are delighted to inform you that your manuscript, "Hepatitis E in Suspected Acute Hepatitis Cases in Brazil: A Comparison with Hepatitis A," has been formally accepted for publication in PLOS Neglected Tropical Diseases.

We have now passed your article onto the PLOS Production Department who will complete the rest of the publication process. All authors will receive a confirmation email upon publication.

The corresponding author will soon be receiving a typeset proof for review, to ensure errors have not been introduced during production. Please review the PDF proof of your manuscript carefully, as this is the last chance to correct any scientific or type-setting errors. Please note that major changes, or those which affect the scientific understanding of the work, will likely cause delays to the publication date of your manuscript. Note: Proofs for Front Matter articles (Editorial, Viewpoint, Symposium, Review, etc...) are generated on a different schedule and may not be made available as quickly.

Soon after your final files are uploaded, the early version of your manuscript will be published online unless you opted out of this process. The date of the early version will be your article's publication date. The final article will be published to the same URL, and all versions of the paper will be accessible to readers.

For Research Articles, you will receive an invoice from PLOS for your publication fee after your manuscript has reached the completed accept phase. If you receive an email requesting payment before acceptance or for any other service, this may be a phishing scheme. Learn how to identify phishing emails and protect your accounts at https://explore.plos.org/phishing.

Thank you again for supporting open-access publishing; we are looking forward to publishing your work in PLOS Neglected Tropical Diseases.

Best regards,

Shaden Kamhawi

co-Editor-in-Chief

PLOS Neglected Tropical Diseases

Paul Brindley

co-Editor-in-Chief

PLOS Neglected Tropical Diseases
